# Motion Anisotropy of Individual, Unconfined Rod-Like-Shaped *Salmonella* Typhimurium Bacteria

**DOI:** 10.1021/acsnanoscienceau.6c00080

**Published:** 2026-09-06

**Authors:** Kevin Diestelhorst, Fereshteh Ghazisaeedi, Anton Klimek, Sebastian Braetz, Karsten Tedin, Roland R. Netz, Marcus Fulde, Stephan Block

**Affiliations:** † Institute of Chemistry and Biochemistry, Department of Biology, Chemistry, Pharmacy, 9166 Freie Universität Berlin, Arnimallee 22, Berlin 14195, Germany; ‡ Research Building SupraFAB, Freie Universität Berlin, Altensteinstr. 23a, Berlin 14195, Germany; § Institute of Microbiology and Epizootics, School of Veterinary Medicine, Freie Universität Berlin, Robert-von-Ostertag-Straße 7-13, Berlin 14163, Germany; ∥ Institute of Microbiology, University of Veterinary Medicine Hannover, Bemeroder Straße 31, Hannover 30559, Germany; ⊥ Veterinary Center for Resistance Research (TZR), Freie Universität Berlin, Robert-von-Ostertag-Straße 8, Berlin 14163, Germany; # Institute of Theoretical Bio- and Soft Matter Physics, Department of Physics, Freie Universität Berlin, Arnimallee 14, Berlin 14195, Germany

**Keywords:** bacterial motility, motional anisotropy, flagella, single-cell tracking, fluorescence microscopy

## Abstract

With the continuous advancement of microscopic imaging techniques, the motion of micro- and nanosized particles can be followed with increasing spatial and temporal accuracy. Analyzing the tracks of single particles enables one to address a broad range of scientific questions, e.g., the dynamics of active and passive (thermally driven) motion or the transport of particles in complex environments. While anisotropic motion, which is exhibited by the vast majority of biologically relevant particles (e.g., bacteria and viruses), has been amply studied in theoretical works, only little experimental data with single-particle resolution have been reported so far. In this study, fluorescence microscopy is used to follow the unconstrained motion of green-fluorescent protein (GFP)-labeled *Salmonella enterica* serovar Typhimurium (*S.* Typhimurium) in bulk. By decomposing the motion of individual bacteria into contributions parallel and perpendicular to the bacterial long axis, we introduce new scores that quantify the degree of motile activity and alignment with high throughput. We find that *S.* Typhimurium possesses a surprisingly broad spectrum of opening angles that range from full alignment of the bacterial long axis with the direction of translational motion to no alignment at all. Applied to mutants, in which motility-related proteins (the flagellins FljB and FliC) have been deleted, we find that wild-type (WT) and FliC-expressing *S.* Typhimurium exhibit very similar motility but differ significantly in the fraction of actively moving bacteria.

## Graphical abstract

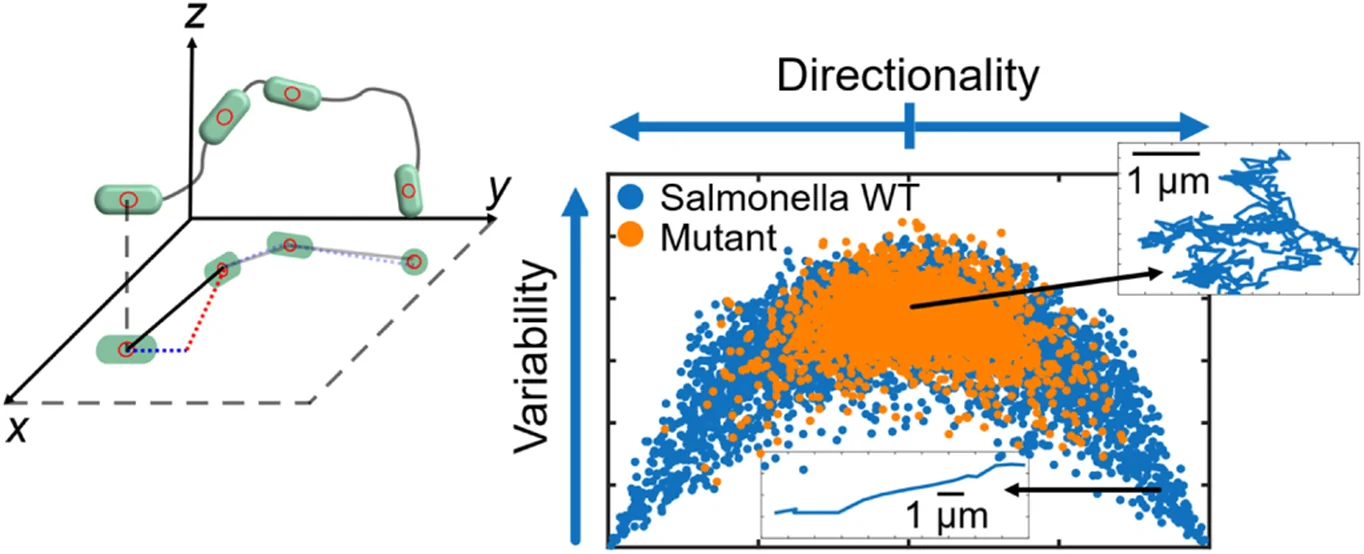

## Introduction

Many bacterial pathogens possess the ability to actively overcome host protective barriers, including the mucus layer that overlays epithelial surfaces of the gastrointestinal tract and other mucosal tissues.^1,2^ Breaching these barriers constitutes the initial event in a complex pathogenic cascade that ultimately facilitates host colonization, subsequent dissemination within the host, and, finally, the establishment of severe disease symptoms. A well-characterized example is infection with pathogenic serovars of *Salmonella enterica*, which are able to pass through the intestinal mucus layer and epithelial barrier, leading to the foodborne disease salmonellosis,^3,4^ one of the major global causes of diarrheal illness.^5^ Members of this species exhibit a rod-shaped morphology and possess 4–6 peritrichous flagella, which drive active movement within the host.^1,2^

Studies on *Salmonella enterica* serovar Typhimurium (*S.* Typhimurium) have shown that flagellar motility is an important determinant of the bacterium’s ability to penetrate mucus and contributes substantially to its infectivity.^2,6–10^ The motion of *S.* Typhimurium is typically described by the run-and-tumble model, in which periods of persistent motion (run period) are transiently interrupted by tumbling events.^11^ Using fluorescently labeled flagella and bacterial bodies, it has been shown that persistent motion is generated if all flagella rotate counterclockwise, causing the flagellar filaments to form a bundle behind the bacterium.^12–14^ This bundle falls apart if one or more of the flagella switch to clockwise rotation, which has been reported to induce a tumbling motion that enables quick changes in the bacterial orientation.^15^ In addition, the motility of *S.* Typhimurium depends not only on the rotational dynamics of the motors but also on structural properties of the flagellar filaments. In particular, the distinct flagellins FljB and FliC, which polymerize to form the flagellar filament, have been shown to influence swimming behavior through differences in filament structure and biomechanics.^15,16^ The motility advantage conferred by the two phase-variable flagellins is strongly context-dependent: while FljB enhances swimming performance under viscous, mucus-like conditions, FliC promotes near-surface motility and epithelial colonization, likely reflecting underlying structural differences in flagellin architecture and filament mechanics.^2,5,7,17^

Bacterial motility can be quantified using optical microscopy combined with single-particle tracking (SPT), allowing for reconstructing of tracks of individual bacteria. Although tracking in all three spatial dimensions has been demonstrated in several reports,^18–26^ the motility of *S.* Typhimurium has been studied mostly in the direct vicinity of an interface (“near-surface swimming”).^2,6,27^ Although this setting enhances the observation time of individual bacteria (and thus the measurement accuracy), the involved confinement may alter the motile behavior and, thus, the bacterial motility. In addition, as *S.* Typhimurium are rod-like-shaped bacteria, their hydrodynamic mobility is anisotropic and differs for motion parallel and perpendicular to their long axis. Hence, at least two translational diffusion coefficients are needed to describe their Brownian motion within simple (*i.e.*, Newtonian) solutions.^28,29^ While anisotropic aspects in the motion of rod-like-shaped objects have been very well covered in theoretical studies,^29–33^ only a few experimental studies with single-particle resolution have been reported for biological particles.^34^ To our knowledge, all bacterial translational diffusion coefficients reported so far have been extracted without explicitly accounting for their anisotropic hydrodynamic cross-section.^35,36^

## Results

In this work, we introduce an SPT analysis framework with high data throughput, which enables us to account for the anisotropic shape of individual bacteria when extracting properties such as translational diffusion coefficients (for random motion parallel and perpendicular to their long axis) or velocity. The feasibility of this framework is demonstrated by tracking the rod-shaped bacterium *S.* Typhimurium, which is a highly relevant enteric pathogen.^3,4^ To this end, we follow the motion of green fluorescent protein (GFP)-labeled *S.* Typhimurium (grown to midexponential phase) in a phosphate-buffered saline (PBS) solution using wide-field (WF) fluorescence microscopy (Figure 1A). As we keep the focal plane fixed during imaging (to maximize data throughput), the recorded image series reflects the two-dimensional projection of the three-dimensional bacterial motion into the focal plane.

**Figure 1:**
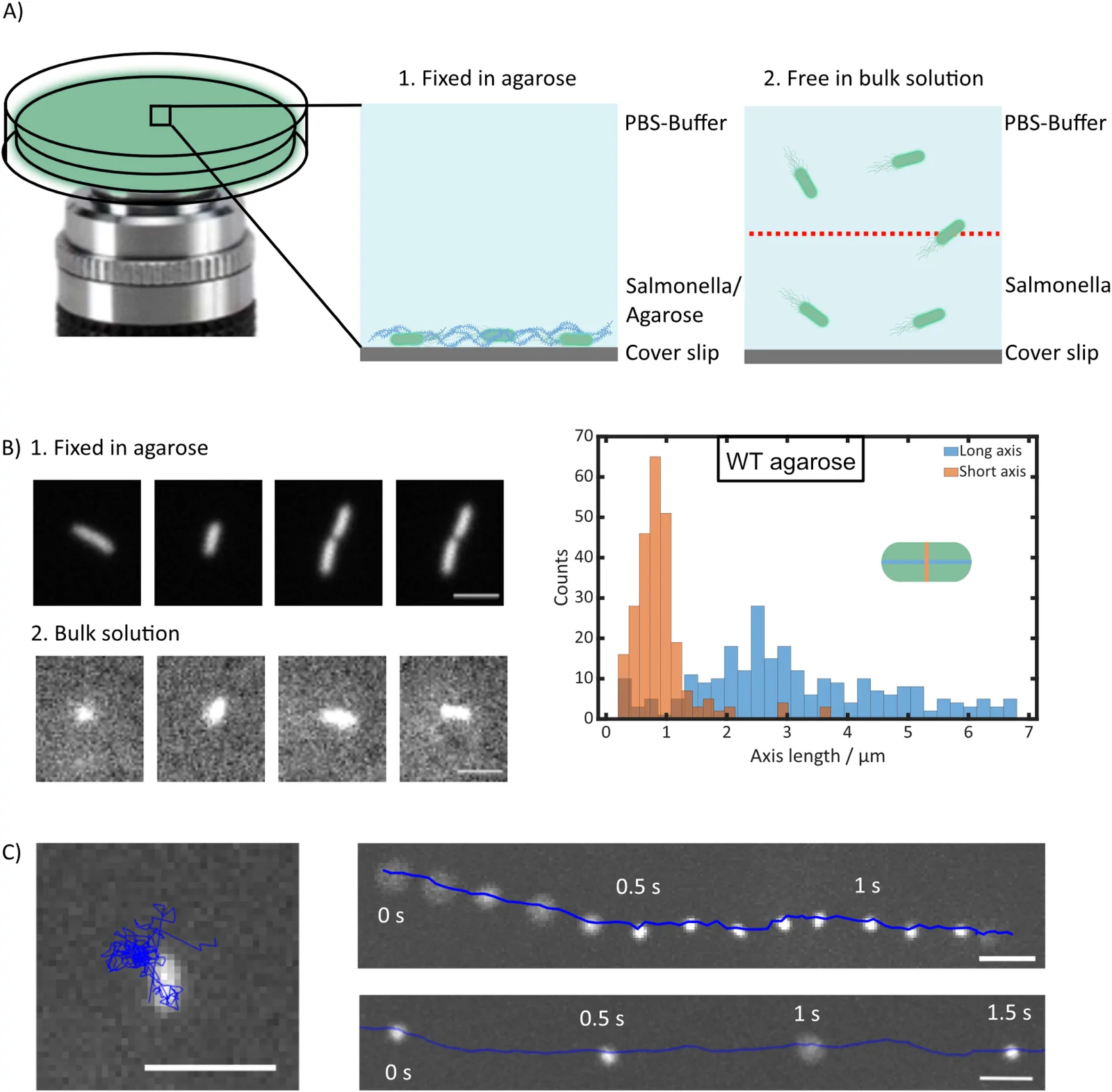
(A) *S.* Typhimurium is imaged using widefield fluorescence microscopy in two settings: (1) after agarose immobilization and (2) during unconstrained motion within PBS-based solutions (dotted line indicates the focal plane). (B) Immobilized *S.* Typhimurium exhibits a rod-like morphology (1). Distributions of the long and short axis lengths are given in the right panel. In contrast, the shape of freely moving bacteria (2) may vary significantly over time, as only the intensity projection into the focal plane is captured. (C) The unconstrained motility covers a broad spectrum, ranging from passive Brownian motion (C, left) to active (directed) motion (C, bottom), including more complex motile patterns (C, top; see also Video S1, Supporting Information). Scale bar: 4 μm.

Prior to the tracking experiments, the size and rod-like (anisotropic) shape of *S.* Typhimurium are determined from microscopic images of agarose-immobilized bacteria, which ensures that all bacteria are aligned with the microscope’s focal plane. In agreement with previous studies,^37,38^ the bacteria exhibit a rod-like shape with an average cross-sectional diameter (short axis) of ∼0.8 μm and an average length (long axis) of ∼3 μm (Figure 1B, right; Figure S2, Supporting Information). Afterward, we image the motion of *S.* Typhimurium in PBS solution (∼30 μm above the interface) and reconstruct tracks of individual bacteria by applying a homemade SPT analysis.^39^ Obtaining high-quality tracks for rod-like objects undergoing unconstrained motion is challenging:

First, as the bacteria rotate in all three dimensions and thus within the focal plane, their apparent shape and size (as visualized by microscopy) may change significantly during the measurement, even when following the same bacterium (Figure 1C). As a consequence, there is no characteristic size scale that describes all bacteria (of apparently small and large size) equally well, which could be used to avoid multiple detections of single bacteria (overcounting) and premature track termination (caused by the too close proximity of two detections) during tracking. Second, while the near-surface swimming of *S.* Typhimurium is reported to follow either random (Brownian) or directed (active) motion,^2,6^ we observe a much higher variance in the motion behavior, covering the entire range between these two limiting cases (Figure 1C), which complicates accurate tracking. In particular, we find that, in the absence of a confining plane, the bacteria may exhibit very complicated tracks, in which the long axis strongly rotates around the center of motion over the entire track (Figure 1C).

In order to refine distortions in single-cell tracking, which are introduced by these complications, we made an in-depth analysis of errors that arise when our tracking analysis framework^39^ is applied to image series of *S.* Typhimurium. In particular, we focused on errors causing premature track termination, as they induce fragmentation of a long track into a series of shorter tracks (reducing the measurement accuracy). By manual side-by-side comparison of true and SPT-derived *S.* Typhimurium tracks, we identified three main reasons for premature track termination, which are either linked to limits in the observation volume (the trivial “out-of-boundary” category) or to transient overcounting of bacteria (“noise-based” and “size-based” fragmentation categories; see Supporting Information Note S1.5 for details). Based on this analysis, we implemented a procedure that automatically assigns all recorded tracks to one of these three categories (Figure 2) and refines the tracks, if possible (details on the assignment scheme and refinement procedure are given in Figures S3–S8, Supporting Information). Application to all data of *S.* Typhimurium (>200,000 bacterial tracks recorded over eight measurement series) indicates that most tracks (52 to 80%, depending on the sample; Table S1, Supporting Information) terminate because the bacteria left the observation volume (“out-of-boundary”), which cannot be avoided nor be refined by postprocessing. For noise-based fragmentation, however, a bacterium is overcounted only in the final frame, which is easily resolved by linking the corresponding tracks. Refinement of bacterial tracks, which have been fragmented due to the extended size of the bacterium (size-based fragmentation category), is more complicated, as directly merging track fragments introduces unphysical jumps in the refined track. In contrast, we determine bacterial displacement by cross-correlating bacterial intensity distributions along all track fragments and generate the refined (nonfragmented) track by integrating these displacements, which significantly improves the track length distribution (Figure 3).

**Figure 2:**
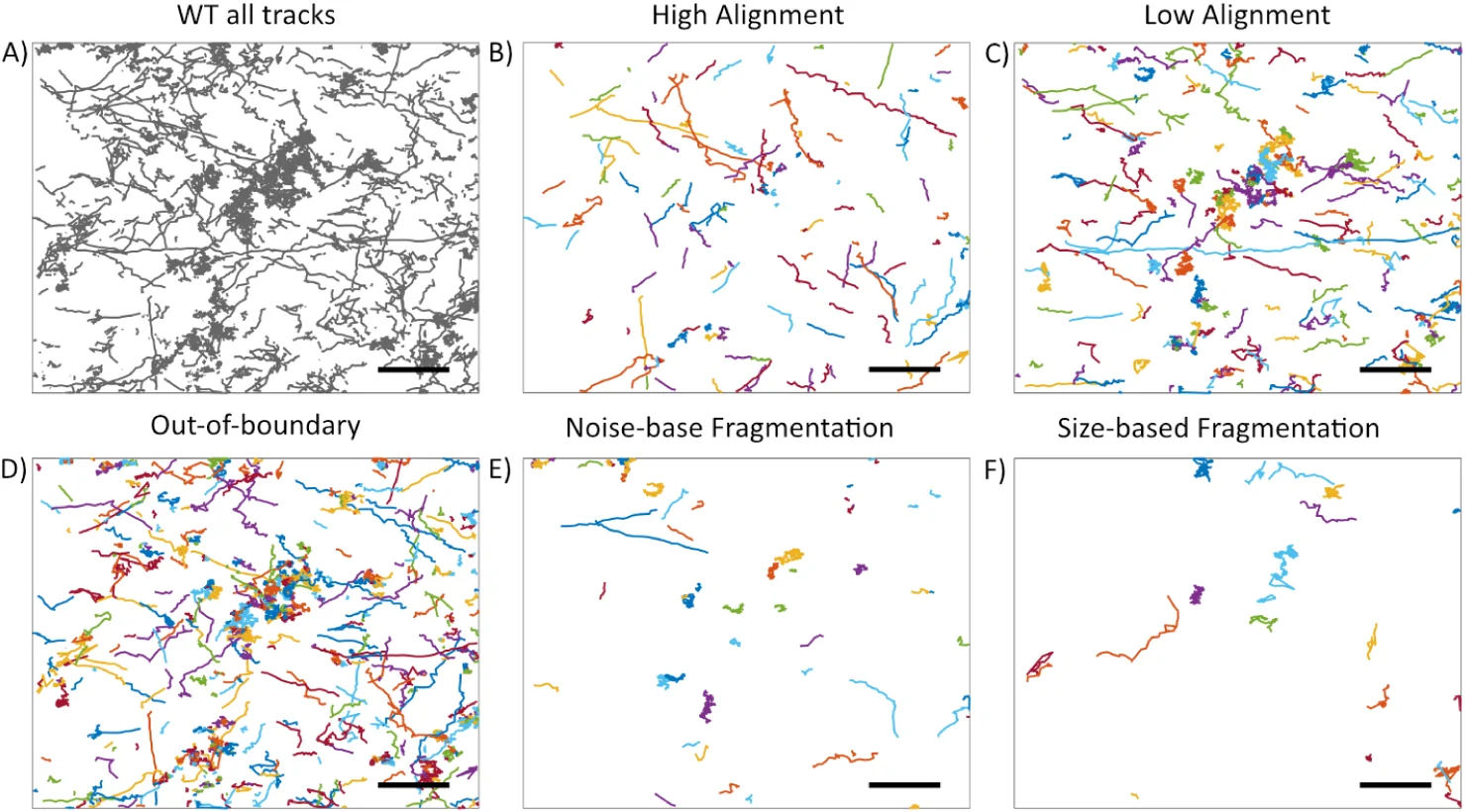
(A) Overlay of all tracks obtained during a representative experiment (*S.* Typhimurium WT). Tracks are classified by an automated categorization scheme based on the alignment of the bacterial long axis and the frame-to-frame displacement (B, C) or the cause of track termination (D-F). Colored tracks indicate the corresponding subsets (the complete ensemble is indicated in gray in A). Interestingly, bacteria with low alignment of the bacterial long axis and frame-to-frame displacement (C) can exhibit persistent tracks of the bacterial center position. Scale bar: 10 μm.

**Figure 3:**
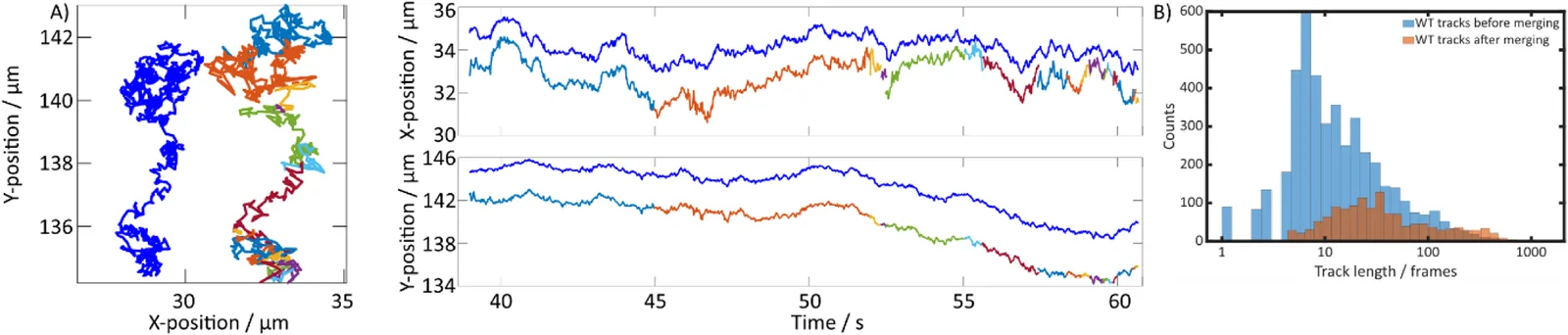
Bacteria with extended intensity distributions often exhibit (size-based) fragmentation due to transient overcounting. (A) The fragmentation is reliably detected and refined by our classification scheme. Fragments (colored curve) and refined track (dark blue curve) are shifted by 5 μm for simplified visualization. (B) Track length distribution before and after refinement.

In the next step, we take advantage of these tracking improvements by analyzing the impact of *S.* Typhimurium’s anisotropic shape on its motility. As the bacteria rotate in all three dimensions, two angles are needed to describe their orientation relative to the focal plane (Figure 4A): *i*) the inclination angle θ between the bacterium’s long axis and the focal plane, and *ii*) the opening angle φ enclosed by the bacteria’s long axis and direction of motion. As the inclination angle θ determines the projected size of the bacterium, it can, in principle, be calculated based on the ratio of projected versus true size of the bacterium.^40^ As the latter varies significantly across the ensemble (Figure 1C), the inclination angle θ cannot be determined reliably in our experiments. In fact, only very few studies have been reported so far that succeeded in extracting θ (typically for only a small number of bacteria),^41,42^ which reflects the general challenge in the determination of the inclination angle when tracking the bulk motion of extended objects.

**Figure 4:**
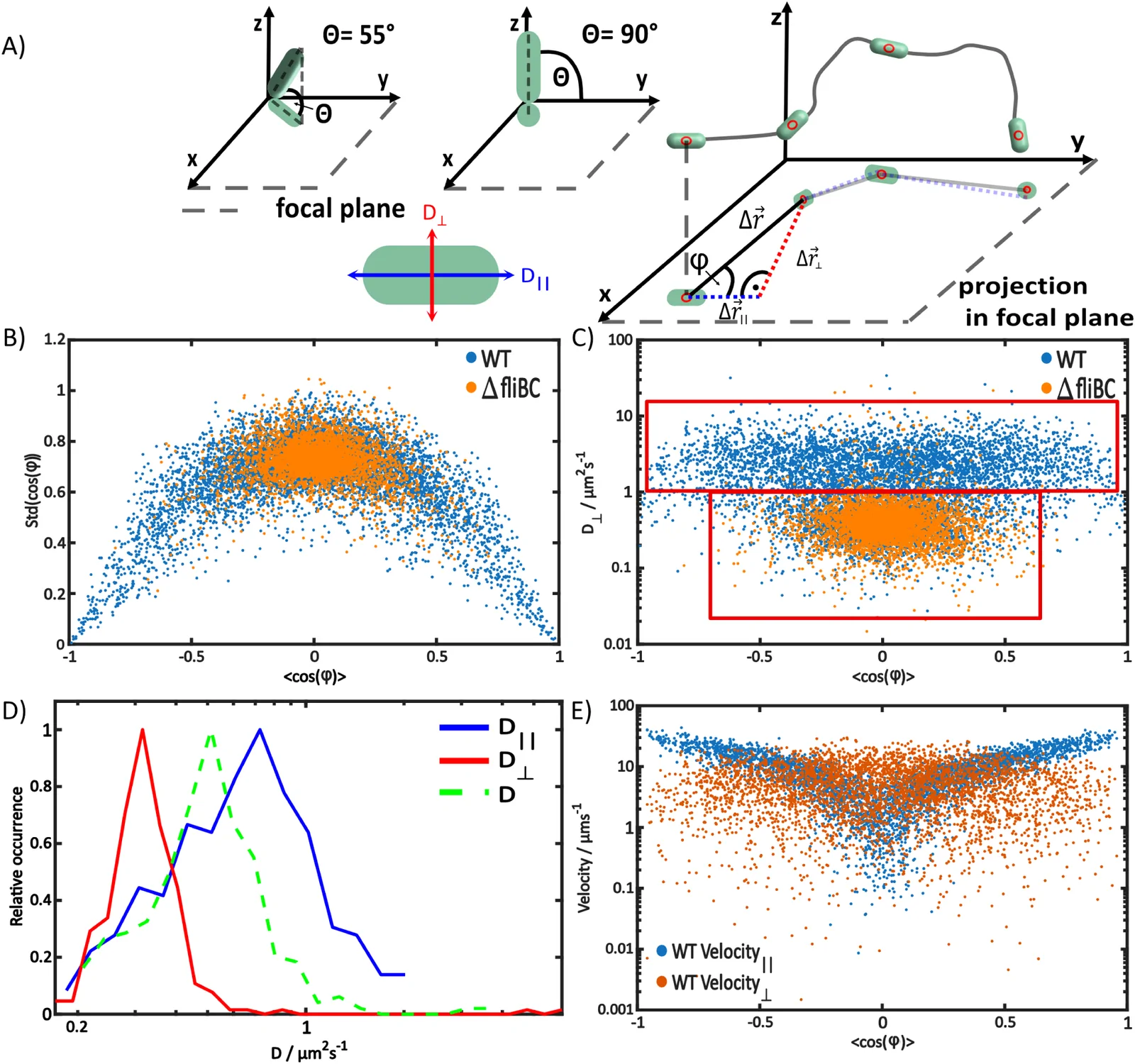
(A) Decomposition of the (projected) frame-by-frame displacement vector Δr⃗ into components parallel (Δr⃗_∥_) and perpendicular (Δr⃗_⊥_) to the bacterial long axis. (B, C) Scatter plot correlating the average value and standard deviation of cos­(φ) and *D*_⊥_ with ⟨cos­(φ)⟩. Each dot corresponds to an individual bacterium (strain as indicated). (D) Diffusion coefficient distributions of passively moving *S.* Typhimurium WT, as calculated from Δr⃗_⊥_ (red line), Δr⃗_∥_ (blue line), and Δr⃗ (green dashed line), respectively (see eqs 2 and 3). (E) Impact of ⟨cos­(φ)⟩ on the average bacterial velocity parallel (*v*_∥_) and perpendicular (*v*_⊥_) to the bacterial long axis (determined for actively moving *S.* Typhimurium WT). While *v*_∥_ strongly depends on the alignment between the bacterial long axis and displacement vector, *v*_⊥_ is independent of ⟨cos­(φ)⟩. Inclusion criteria: (B, C) track length > 5 frames; (D) inactive population, track length > 50 frames, aspect ratio ≥ 1.75; (E) active population (*D*_⊥_ > 1 μm^2^/s), track length > 5 frames.

Nevertheless, we successfully extract the opening angle φ from the coordinates of two consecutive frames (*i.e.*, the displacement vector), provided that the direction of the long axis can be resolved. This is only possible for bacteria that exhibit anisotropic intensity distributions (*i.e.*, at sufficiently small inclination angles). We therefore determine the opening angle φ for all bacteria that exhibited an aspect ratio (AR, ratio of long versus short axis) exceeding 1.75, corresponding to inclination angles < ∼55°, and the resulting tracks contain information on the frame-to-frame displacement vector Δr⃗ and the of the bacterial long axis. We combine both quantities (Figure 4A) and decompose the two-dimensional displacement vector Δr⃗ into (one-dimensional) displacements parallel and perpendicular to the long axis, Δ*r*_∥_ and Δ*r*_⊥_, respectively. From these quantities, the average velocity parallel (*v*_∥_) and perpendicular (*v*_⊥_) to the bacterial long axis is calculated according to

1 $$v_{∥}=\frac{\left\langle \Delta r_{∥}\right\rangle }{\Delta t}\;\mathrm{and}\;v_{⊥}=\frac{\left\langle \Delta r_{⊥}\right\rangle }{\Delta t}$$

(Δ*t* denotes the time span between two consecutive frames and the brackets indicate the average value of Δ*r*_∥_ or Δ*r*_⊥_ taken over the entire bacterial track). Analogously, we quantify the short-term fluctuations of the bacterial center position in these two directions according to

2 $$D_{∥}=\frac{var\left(\Delta r_{∥}\right)}{2\Delta t}\;\mathrm{and}\;D_{⊥}=\frac{var\left(\Delta r_{⊥}\right)}{2\Delta t}$$

(with *var*(*x*) = ⟨*x* ^2^⟩ – ⟨*x⟩* ^2^ denoting the variance taken over the entire track). As the involved displacements have been derived from the projection of three-dimensional bacterial motion into the focal plane. Equations 1 and 2 yield quantities that correspond to projections of the underlying 3D quantities. For passively diffusing bacteria, the positional fluctuations parallel and perpendicular to the bacterial long axis are expected to follow the corresponding Stokes–Einstein diffusion coefficients.^32,33^ In this case, the quantity *D*_∥_ corresponds to the projection of the translational diffusion coefficient for random motion parallel to the long axis, whereas *D*_⊥_, due to the rotational symmetry of rod-like-shaped objects, is expected to be equivalent to the translational diffusion coefficient for random motion perpendicular to the long axis. For actively moving bacteria, this relationship breaks down, and the long-term diffusion coefficients depend on multiple quantities, such as the durations of run- and tumble-periods, as well as the velocity during persistent motion.^43^

Nevertheless, we extract these quantities for all tracks that covered at least 5 frames, and the involved decomposition goes beyond related studies on bacterial motility,^35,36^ in which the translational diffusion coefficient *D* of a bacterium is calculated using

3 $$D=\frac{\left\langle \Delta {\mathrm{r⃗}}^{2}\right\rangle }{4\Delta t}$$

(not accounting for anisotropy). In addition, we extract, for all opening angles φ of an included track, the mean value and standard deviation (*std*) of cos­(φ), which quantifies the variability of the alignment between the long axis and displacement vector over the track. Figure 4B shows the corresponding scatter plot for wild-type (WT) *S.* Typhimurium as well as a mutant that is unable to form functional flagella (Δ*fliBC*) and is therefore incapable of active motion. All related scatter plots are reproduced in Figure S12, Supporting Information.

Initially, we assumed that actively moving bacteria would predominantly move in the direction of their long axis, as this minimizes the hydrodynamic cross-section of the bacterium. This assumption is in line with tracking experiments involving *Escherichia coli* (*E. coli*), which showed that the bacterial long axis “wobbles” around the direction of translational motion with relatively small opening angles (φ < 25^°^).^15^ Based on these measurements, we expected that actively moving *S.* Typhimurium bacteria would exhibit notable alignment and thus little variation of φ along an individual track. In contrast, a much higher variability of φ is expected for passively diffusing (*i.e.*, randomly moving) bacteria. Hence, with increasing alignment (⟨cos­(φ)⟩ approaching ±1 in Figure 4B), the standard deviation of cos­(φ) is expected to decrease. This behavior is indeed observed for *S.* Typhimurium WT (Figure 4B), whereas the data of the passively diffusing Δ*fliBC* mutant forms a point cloud centered around ⟨cos­(φ)⟩ ≈ 0 and *std*(cos­(φ)) ≈ 0.7. Hence, any data point outside of the Δ*fliBC* point cloud is indicative of active motion (which was confirmed by a manual inspection of the corresponding tracks) and suggests using ⟨cos­(φ)⟩ as a proxy to identify active motion.

However, we also find a significant number of tracks, which show clear features of active motion (*i.e.*, deterministic rotation and/or translation) but whose ⟨cos­(φ)⟩ scores fall within the area of the Δ*fliBC* point cloud. Examples for such tracks are given in the Supporting Videos S1 –S3, all of which show no notable alignment (*i.e.*, a ⟨cos­(φ)⟩ value close to zero). Hence, for tracks located in this area, it is not possible to directly conclude their type of mobility (active vs. passive). In the next step, we investigate the impact of bacterial motility on the diffusive properties *D*_⊥_ and *D*_∥_ by correlating ⟨cos­(φ)⟩ with *D*_⊥_ (Figure 4C) and *D*_∥_ (Figure S13 and S14, Supporting Information), respectively. Interestingly, two distinct areas are resolvable: *i*) one point cloud centered around *D*_⊥_ values of ∼0.3 μm^2^/s and *ii*) an extended band located above *D*_⊥_ values of ∼1 μm^2^/s. The data of the point cloud exhibit only ⟨cos­(φ)⟩ values close to zero, implying that this region of the scatter plot corresponds to passively diffusing bacteria. This notion is confirmed by overlaying the Δ*fliBC* mutant (Figure 4C), which lacks the extended band and exhibits only the point cloud (covering exactly the same area as passively diffusing *S.* Typhimurium WT).

The extended band with *D*_⊥_ values >1 μm^2^/s therefore corresponds to actively moving bacteria, and the *D*_⊥_ vs. ⟨cos­(φ)⟩ scatter plot provides a convenient means to distinguish passively and actively moving bacteria (which we confirmed by visual inspection). This observation implies, however, that actively moving *S.* Typhimurium WT exhibits a broad spectrum of opening angles that range from full alignment of the bacterial long axis with the direction of translational motion to essentially no alignment at all. In particular, low alignment can only be observed if *S.* Typhimurium WT exhibits much larger opening angles than those reported for peritrichously flagellated bacteria.^15^ To confirm this notion, we performed high-speed SPT experiments with an acquisition rate of 250 fps, which enables the analysis of bacterial motion in more detail. These measurements indeed reveal that *S.* Typhimurium WT can adopt very large opening angles; three representative examples with values exceeding 110° are shown in Figure S16 and in Videos S4 -6 (Supporting Information).

For passively moving *S.* Typhimurium, we investigate the impact of shape anisotropy on the random motion by comparing their *D*_∥_ and *D*_⊥_ distributions (Figure 4D; Figure S10, Supporting Information). To improve the measurement accuracy, only tracks with AR-values exceeding 1.75 and covering more than 50 frames are included in this analysis. For *S.* Typhimurium WT, we find median values of 0.64 and 0.32 μm^2^/s for *D*_∥_ and *D*_⊥_, respectively, which are in good agreement with theoretical estimates for the diffusion of rod-like objects^32–34^ and are on the same order of magnitude as reported for spherical particles.^44,45^ Furthermore, an analysis of the corresponding mean squared displacement curves (Figure S11, Supporting Information) reveals a linear lag time dependence and very similar *D*_∥_ and *D*_⊥_ values as the frame-by-frame analysis, indicating that the positional fluctuations of passively moving *S.* Typhimurium WT indeed follow Brownian motion. The shift between the *D*_∥_ and *D*_⊥_ distribution is reasonable, as the hydrodynamic friction experienced by such rod-like-shaped objects is larger for motions perpendicular than parallel to the long axis. We find that the *D*_∥_ distribution is significantly broader than the *D*_⊥_ distribution, which is in line with the notion that *D*_∥_ corresponds to the projected diffusion coefficient and therefore depends on two properties: the corresponding Stokes–Einstein diffusion coefficient and inclination angle *θ.* In contrast, *D*_⊥_ is a measure for the diffusion coefficient perpendicular to the long axis, which is a consequence of the rotational symmetry of rod-like object (as discussed below eq 2). The *D* distribution (eq 3), which does not account for anisotropy, is located in betweenthe *D*_∥_ and *D*_⊥_ distribution.

Interestingly, we find that actively moving *S.* Typhimurium WT exhibit much higher *D*_⊥_ values than passively diffusing bacteria (∼2.4 μm^2^/s vs. ∼0.4 μm^2^/s, see Table S2, Supporting Information), indicating that random fluctuations of the bacterial center position increase significantly beyond Stokes–Einstein in the presence of active motion. Although active motion is the clear driver of this increase, the *D*_⊥_ values do not show any resolvable dependence of the alignment between the bacterial long axis and frame-to-frame displacement ⟨cos­(φ)⟩. This observation indicates that there is no significant difference between the *D*_⊥_ values of bacteria that exhibit almost straight tracks compared to those which lack such high alignment (Figure S15, Supporting Information) and implies that imperfections in the description of the actively moving bacteria are not the only driver for the enhanced *D*_⊥_ values observed.

For actively moving bacteria (*D*_⊥_ > 1 μm^2^/s; Figure 4C, upper red box), we correlate the alignment, ⟨cos­(φ)⟩, with the average velocity parallel (*v*_∥_) and perpendicular (*v*_⊥_) to the long axis at the level of individual bacteria (eq 1 and Figure 4E). While there is no notable impact on *v*_⊥_, we observe a systematic increase of *v*_∥_ with increasing alignment. The mode of the *v*_∥_ distribution is located at ∼7 μm/s for *S.* Typhimurium WT, which is smaller in comparison to related studies^2,6,9,35,46,47^ and is attributed to the fact that all actively moving bacteria are included in this analysis (including bacteria that exhibit little alignment in their motion). This notion is supported by the observation that *v*_∥_ approaches the literature value of ∼35 μm/s for bacteria, for which the bacterial displacement and long axis are fully aligned.^2,6,9,35,46,47^

Motility in *S.* Typhimurium is driven by long helical filaments that are connected to the bacterial cell envelope via a flexible hook, which, in turn, connects to a basal body embedded in the membrane and housing the rotary motor that generates torque.^5^ This motor–hook–filament assembly constitutes the bacterial flagellum, a complex nanomachine responsible for propulsion. In the next step, we investigate how the flagellin composition of the flagellar filament influences *S.* Typhimurium motility. The filament is assembled from thousands of subunits of the globular protein flagellin. *S.* Typhimurium can express two antigenically distinct flagellins, FljB and FliC, through a well-characterized phase-variation mechanism.^2,5,7,48^ Although both variants occur within the bacterial population, an individual bacterium expresses only one flagellin variant at a time, such that all of its flagella consist exclusively of either FljB or FliC subunits. To elucidate the specific contribution of each flagellin type to *S.* Typhimurium motility, we used mutants in which the genes encoding FljB, FliC, or both flagellins were deleted (referred to as Δ*fljB*, Δ*fliC,* and Δ*fliBC*, respectively).

Application of our methodology reveals that the inactive population of *S.* Typhimurium WT and the mutants exhibit very similar *D*_⊥_ distributions (peak centered around ∼0.4 μm^2^/s in Figure 5A), indicating that all strains exhibit similar length distributions. Differences are found, however, in the number ratio of actively versus passively moving bacteria, which is largest for WT, decreases for Δ*fljB* and is essentially zero for Δ*fliC* and Δ*fliBC*. A similar behavior is found for the *v*_∥_ distributions (Figure 5B), which also display peaks corresponding to actively (*v*_∥_ > 5 μm/s) and passively moving bacteria, and a decrease in the number ratio of actively versus passively moving bacteria in the same order as observed for the *D*_⊥_ distributions.

**Figure 5:**
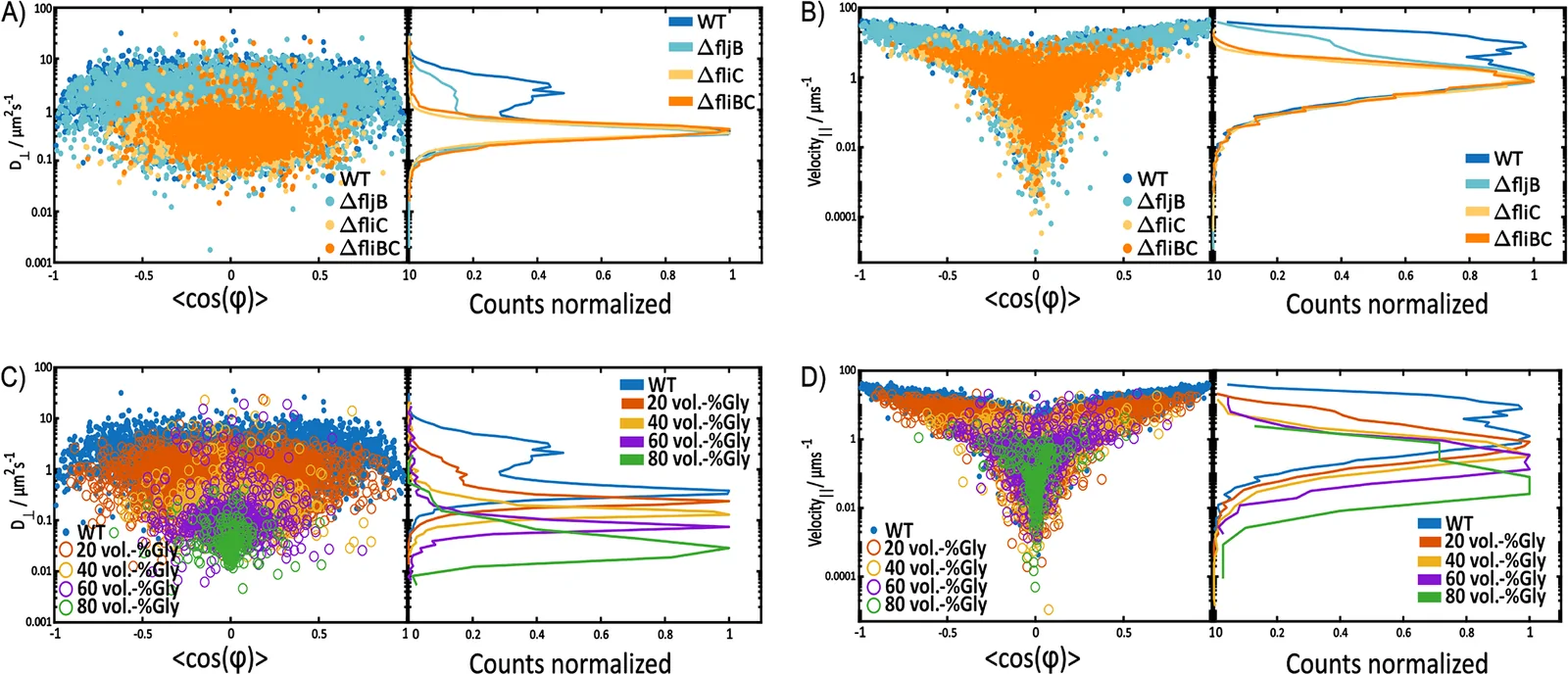
(A, B) Impact of *S.* Typhimurium strain and (C, D) solvent viscosity on (A, C) *D*_⊥_ and (B, D) *v*_∥_. Inclusion criterion: track length > 5 frames.

Finally, the impact of the solution’s viscosity on bacterial motility is addressed by following the motion of *S.* Typhimurium WT in buffer-glycerol mixtures (Figure 5). As expected, increasing the viscosity of the solution causes the *D*_⊥_ distribution to shift to lower values, whereas the observed shift is (within experimental error) in quantitative agreement with the increase in viscosity (Table S2, Supporting Information). Nevertheless, quantifying the impact of viscosity on the velocity is complicated by the observation that, with the increasing volume fraction of glycerol (i.e., increasing viscosity), the fraction of actively moving bacteria (across the ensemble) decreases. As a consequence, only up to 40 vol % glycerol is it possible to extract the velocity distribution (Figure 5C and D), whereas for higher glycerol volume fractions, the measurement statistics only permit the extraction of the ensemble-averaged velocity value. Nevertheless, this measurement shows that increasing the viscosity by a factor of ∼2 causes the *D*_⊥_ and *v*_∥_ distributions to shift to lower values, which is in line with the expected increase in hydrodynamic friction.

## Conclusions

Motility is a key determinant of bacterial pathogenicity and survival. Understanding motility at the single-cell level is essential for elucidating how individual bacteria behave within a population, providing critical insight into the early steps of host invasion and infection. Optical microscopy, combined with SPT, is one of the most widely used approaches for describing and quantifying bacterial motion. In this study, optical microscopy is employed to follow the unconstrained motion of *S.* Typhimurium in bulk solution with high throughput. Home-made SPT is applied to reconstruct tracks at the level of individual bacteria that combine information on the bacterial center position with bacterial orientation. This information enables us to quantify motile properties, such as the frame-to-frame velocity and fluctuations of the center position, parallel and perpendicular to the bacterial long axis, and hence account for anisotropy in the quantification of bacterial motility. In contrast to related experimental studies, in which only a single (isotropically averaged) translational diffusion coefficient is provided per bacterium, the presented approach enables the decomposition of fluctuations of the bacterial center position into two components, being either parallel (*D*_∥_) or perpendicular (*D*_⊥_) to the bacterial long axis. Furthermore, it also quantifies the opening angle, which is enclosed between the bacteria’s long axis and displacement vector at the level of individual bacteria. Our analysis shows that actively moving bacteria exhibit significantly larger *D*_⊥_ values than passively diffusing ones, which provides a convenient means to determine the active population in the bacterial ensemble. In addition, we introduce the alignment between the bacterial long track and the resulting motion as a new motility score to analyze motile patterns and show that actively moving bacteria cover all levels of alignment. In this way, we demonstrate that *S.* Typhimurium exhibits a much wider range of opening angles than reported in related species and that both quantities have to be taken into account to comprehensively describe bacterial motion. Taken together, we present an SPT-based framework that allows to quantify anisotropic motility of freely moving (*i.e.*, unconstrained) bacteria with high data throughput, providing an improved methodology for dissecting bacterial swimming behavior at the single-cell level.

## Methods

### Cultivation of Bacteria

*Salmonella* Typhimurium and its respective flagellin-deficient mutant strains (MvP1756 (Δ*fliC*), MvP1757 (Δ*fljB*), and MvP1761 (Δ*flijB*/*fliC*)) carrying the constitutively GFP-expressing plasmid (pFPV25.1)^49^ were grown overnight on Luria–Bertani (LB) agar plates (LB Lennox, Carl Roth) at 37 °C. Single colonies from the overnight cultures were inoculated into LB broth for another 24 h. Overnight cultures were then diluted 1:20 into fresh LB medium and incubated for an additional 4 h, until reaching an OD_600_ of approximately 0.5 (mid-logarithmic growth phase). Where appropriate, 100 μg/mL of ampicillin (Carl Roth) or 50 μg/mL of kanamycin (Sigma-Aldrich) was added to the cultures for selection. Cultures that reached the mid-log growth phase were washed in phosphate-buffered saline (PBS) and resuspended in PBS, then adjusted to an optical density at 600 nm of 0.5 (approximately 2.5 × 10^8^ bacteria/mL). This suspension served as the standardized working stock from which the experimental dilutions were prepared. For assessing the effect of solution viscosity on bacterial motility, glycerol (Carl Roth, Glycerin ROTIPURAN® ≥99.5%, p.a., water-free) was used to prepare glycerol-water mixtures with defined bulk viscosities.

### Fluorescence Microscopy

Wide-field microscopy was performed at room temperature using a Leica DMI6000 B inverted microscope (Leica, Wetzlar, Germany) equipped with an HCX PL APO 63x/1.40 OIL (Leica, Wetzlar, Germany) immersion objective and a Prime BSI sCMOS camera (Teledyne Photometrics). To quantify bacterial motility in PBS, a resuspension of *S.* Typhimurium of 300 μL (OD 0.8) or 600 μL (OD 0.5) was diluted with PBS to obtain a final volume of 2 mL. For the glycerol measurements, 300 μL (OD 0.8) of *S.* Typhimurium was diluted to 2 mL with glycerol-PBS mixtures (as indicated in the manuscript). Afterward, the final solution (0.75 × 10^8^ bacteria/mL) was added into an Ibidi-μ-Dish 35 mm, high. Microscopy recordings were made with the ImageJ plugin Micro-Manager 2.0.^50^ Each individual time series comprised 20,000 frames that were recorded using the full sensor (1024 pixels × 1024 pixels) with a 2 × 2 binning, a 10 ms exposure time with no delay between consecutive frames, resulting in a temporal resolution of 43 fps (frames per second). Image series with higher temporal resolution were recorded using a hardware-defined region of interest of 128 pixels × 128 pixels with a 2 × 2 binning, which resulted in a 250 fps acquisition rate (4 ms exposure time with no delay between consecutive frames).

### Single-Cell Tracking

All data analysis is performed using custom-made scripts written in MATLAB® (MathWorks, Natick, MA).^39^ Particles are detected by a local maximum approach combined with intensity thresholding. The cutoff value for the thresholding is manually determined by visual inspection of the raw image series using ImageJ and allows to differentiate between true detection events and local maxima of the background signal. The brightest pixel in a defined region of interest (7 × 7 window) for every event is chosen as the particle coordinates in pixel resolution. Particle linking is implemented using the local nearest-neighbor linking algorithm.^51^ Spatial resolution is enhanced below the pixel limit by using a 2D Gaussian function. In addition, systematic drifts (*e.g.*, convection-induced collective motion of the bacteria) are adjusted by a drift compensation procedure. This drift correction step calculates the frame-by-frame displacement in the *x* and *y* directions by analyzing tracks with a sufficient number of frames and sufficient localization quality, followed by the calculation of the median of the *x*- and *y*-displacement for each frame from the corresponding frame-by-frame displacements. These frame-dependent median values (in *x*- and *y*-directions) are subtracted from all tracks.^52^ Subsequently, in order to obtain accurate and highly reliable trajectories, the classifier described in detail in Supporting Section S1.3 is used to automatically spot and correct common detection and linking errors (*e.g.*, noise-based fragmentation).

## Supplementary Material















## Notes

Additional experimental details, materials, and methods, including (i) size distributions of the *S.* Typhimurium strains used in the study, (ii) the bacterial tracking and refinement of bacterial tracks, and (iii) the distributions of tracking-related bacterial properties (PDF)
Representative videos showing the motile behavior of individual bacteria recorded at 43 fps (AVI, AVI, and AVI) and at 250 fps (AVI, AVI, and AVI)
