# Supplementary material for: Motion Anisotropy of Individual, Unconfined Rod-Like-Shaped Salmonella Typhimurium Bacteria
Source: ACS Nanosci Au. 2026 Sep 6;6(5):913–22. doi: 10.1021/acsnanoscienceau.6c00080 (PMC13693235; doi:10.1021/acsnanoscienceau.6c00080)
Supplement: Supplementary file 1 [file ng6c00080_si_001.pdf]

Supporting information of the manuscript

Motion anisotropy of individual, unconfined rod-like-shaped *Salmonella* Typhimurium bacteria

Kevin Diestelhorst[1,2], Fereshteh Ghazisaeedi[3,4], Anton Klimek[6], Sebastian Braetz[3], Karsten Tedin[3], Roland R. Netz[6], Marcus Fulde[3,4,5], Stephan Block[1,2]

[1] Institute of Chemistry and Biochemistry, Department of Biology, Chemistry, Pharmacy, Freie Universität Berlin

[2] Research Building SupraFAB, Freie Universität Berlin, Altensteinstr. 23a, 14195 Berlin, Germany

[3] Institute of Microbiology and Epizootics, School of Veterinary Medicine, Freie Universität Berlin, Germany

[4] Institute of Microbiology, University of Veterinary Medicine Hannover, Hannover, Germany

[5] Veterinary Center for Resistance Research (TZR), Freie Universität Berlin, Berlin, Germany

[6] Institute of Theoretical Bio- and Soft Matter Physics, Department of Physics, Freie Universität Berlin

S1. Methods

S1.1. Fluorescence microscopy

The optical resolution of the microscope was quantified by imaging a calibration ruler slide (Figure S1). The distance between two ruler ticks corresponds to 0.01 mm and appeared to be separated by 98 pixels in the images, yielding an effective pixel size of 0.102 µm per pixel at 1x1 binning. The effective field of view in the measurements is therefore 215 µm x 215 µm.

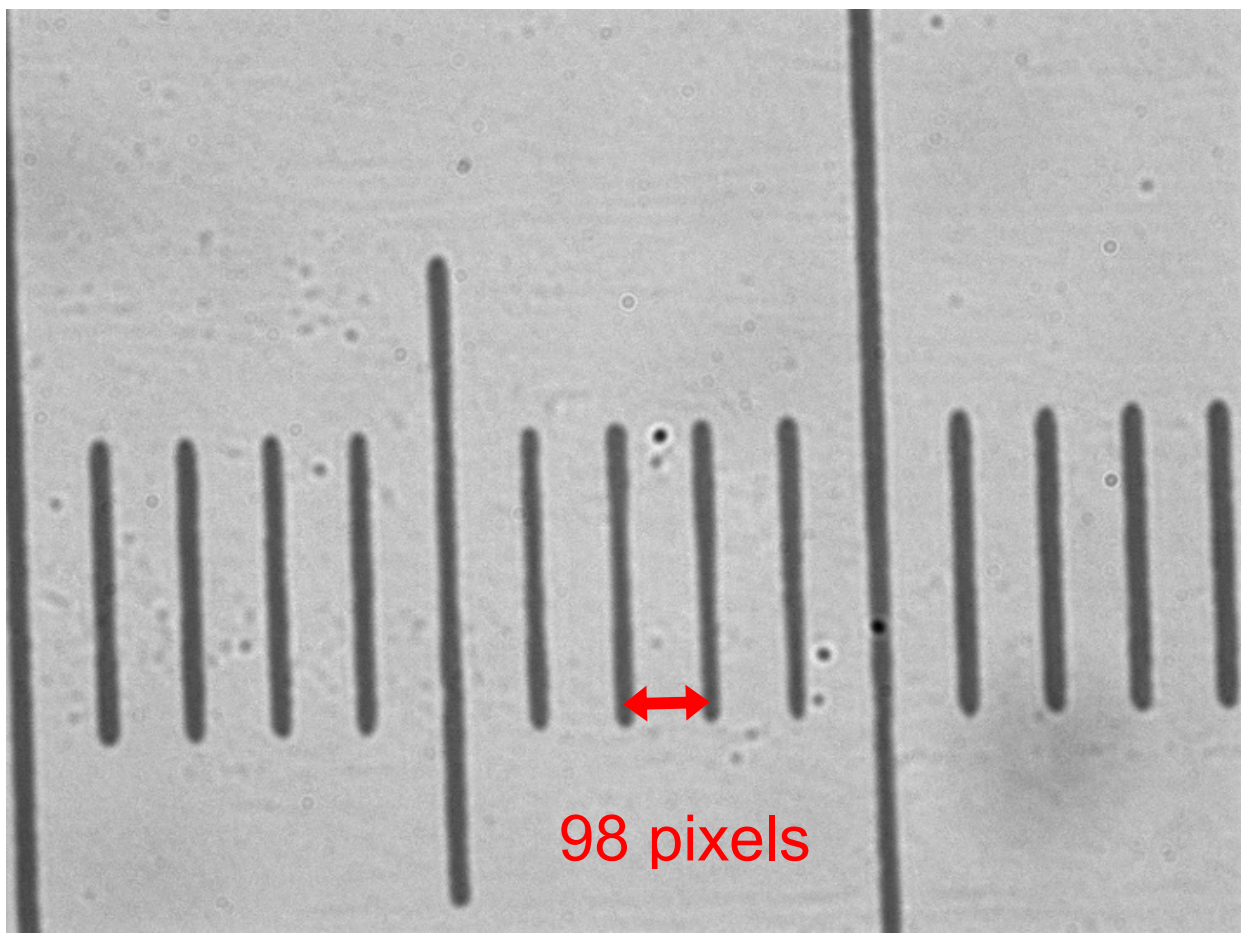

Figure S1: Ruler-based calibration of the pixel size (recorded using a 1x1 binning). One division equals a physical distance of 0.01 mm.

S1.2. Size distributions of S. Typhimurium immobilized on agarose

To determine the size values of the *S.* Typhimurium, bacteria were fixed on an agarose gel at a glass surface. In this setting, the bacteria are oriented parallel to the focal plane and the long (major) and short (minor) axis can be determined (Figure S2).

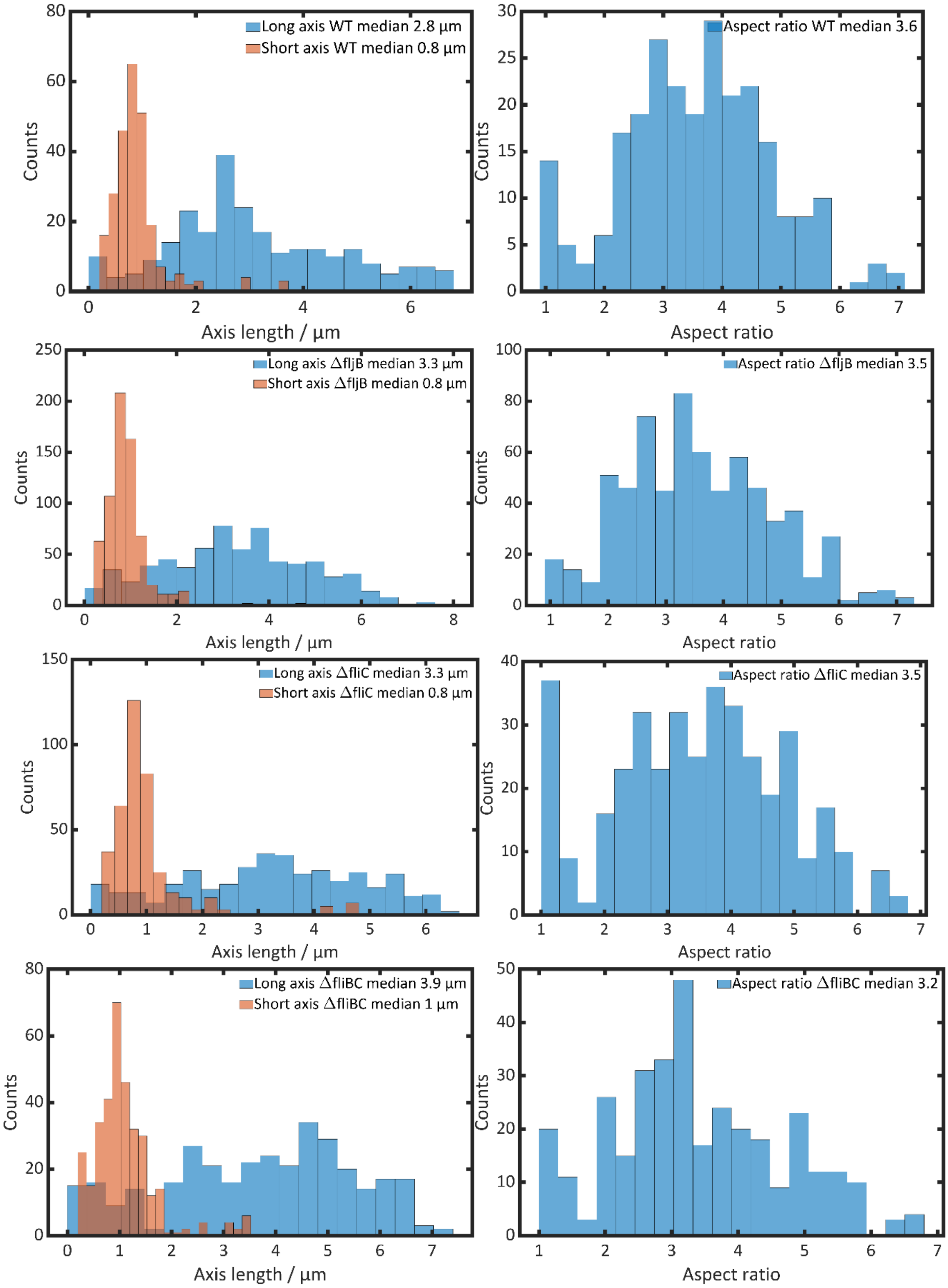

Figure S2: Size distribution (left) and aspect ratio (right) of S. Typhimurium *(WT and mutants) immobilized on agarose. Shown are distributions that pool the data of three (WT, ΔfliC), five (ΔfljB), or six (ΔfliBC) different spots.*

S1.3 Automatic classification and refinement of bacterial tracks
To improve the quality of the bacterial tracks, we analyzed typical errors that are made by the previous analysis steps when tracking *S.* Typhimurium. In particular, we focused on reasons that led to premature track termination, which we identified by a manual side-by-side comparison of true and SPT-derived *S.* Typhimurium (Figure S3):

As the observation volume is fixed (215 µm x 215 µm x ~3 µm) during the measurement, bacteria that either move out of the field of view or out of the focal plane can no longer be tracked. Tracks of this (first and trivial) "out-of-boundary" category terminate due to limitations of the observation volume and cannot be refined. Another source of premature track termination is caused by a transient overcounting (*i.e.*, the same bacterium is detected multiple times in a single frame), which can be refined once identified. We observed that such overcounting can originate from random intensity fluctuations, which eventually distorted the intensity profile such that multiple pixels fulfill the inclusion criteria for a detection (second, "noise-based fragmentation" category). In addition, we often observed transient overcounting for bacteria of large apparent size (third, "size-based fragmentation" category), which are typically unfocussed bacteria (being too far away from the focal plane) or bacterial doublets (Figure 1B, main manuscript).

In order to refine tracks that were subject to overcounting, we established a data-driven approach that automatically identify the corresponding tracks: To this end, each detected track is assigned an ID and tracks are included in further data processing, if the track length exceeds 5 frames. For each included track, its *x* and *y* position and intensity value are determined in the terminating frame. Furthermore, the distances to all neighboring tracks are calculated for the terminating frame as well as the entire track duration. From this data, the track is identified, which exhibits the smallest distance to the analyzed track over the track duration (the corresponding ID and distance as well as the number of overlapping frames are denoted as ID*, Δxy*, and ΔN* in Figure S7) and in the terminating frame (ID**, Δxy** and ΔN** in Figure S7), respectively.

After having collected this data for all included tracks, their reason of track termination is determined as follows: The spatial (x and y) position and the intensity in the terminating frame are used to identify tracks, which terminated as the corresponding bacteria moved "Out-of-boundary" during the observation. Tracks were included in this category, if their spatial position less than 5 pixels away from one of the edges of the field of view or if their intensity value less than 35 counts higher than the intensity threshold used in the detection process (see Figure S6). Tracks in this category were not further refined. The outcome of this classification revealed that the majority tracks (52 to 80 %, depending on the sample; Table S1) terminated because the bacteria left the observation volume ("out-of-boundary"), which cannot be avoided nor be refined by post-processing.

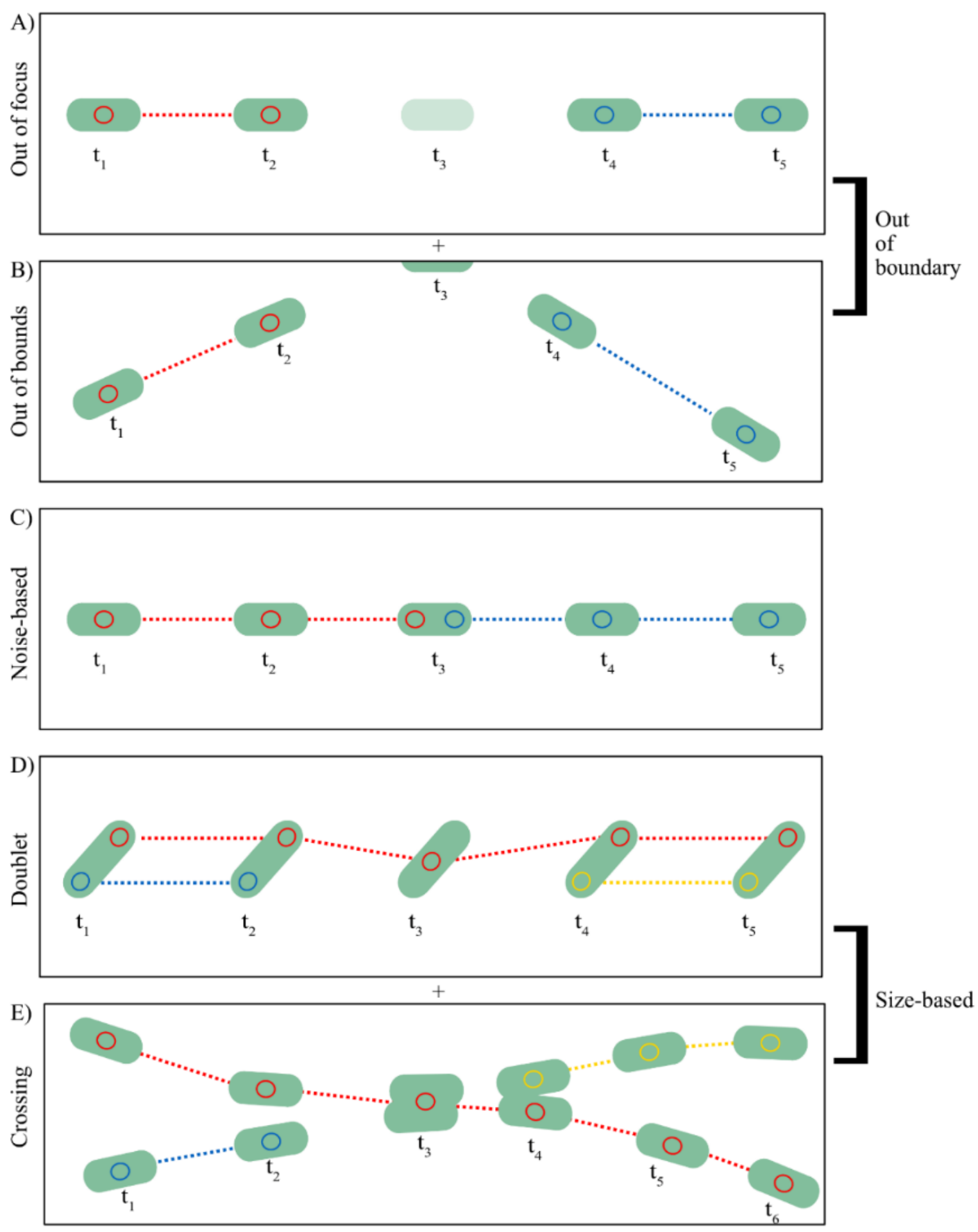

Figure S3: Illustration of various reasons that can lead to track termination. In each panel (A-E) S. Typhimurium are shown by different time steps t_n. Detection events are symbolized by an open circle, whereas the color indicates the track to which the particular event belongs to. A) Out of focus motion of the bacterium causes its intensity to drop below the inclusion threshold. B) Complete or partial leaving of the observation area (field of view). C) Intensity noise may cause two pixels of a bacterium to fulfil the inclusion criteria, leading to a transient double detection of the same bacterium. D) Multiple detection of spatially extended bacteria yield multiple tracks per bacterium, which fragment at frames, in which detection events of the bacterium are lost (e.g., due to reorientation of the bacterium perpendicular to the focal plane). E) The tracks of mobile bacteria may cross with other bacteria, during which both bacteria generate only a single detection event.

The remaining fraction of tracks (20- 48%) exhibited fragmentation due to transient overcounting, which we refined the tracks as follows (Figure S4): For noise-based fragmentation, only the final frame is affected, which is easily resolved by linking the corresponding tracks. In contrast, refinement in the size-based fragmentation category is more complicated, as the track fragments typically reflect individual parts of the same bacteria and a direct merging of them introduces unphysical jumps. We address this challenge using a cross-correlation approach, in which the bacterial displacement is determined by cross-correlating bacterial intensity distributions originating from subsequent frames. Integration of these displacements then reconstructs the full non-fragmented track, which significantly improves the track length distribution (Figure 3).

The assignment to the remaining categories was done based on the distances to the neighboring tracks (Figure S7). Tracks were assigned to the category "size-based fragmentation" if, over the course of the track, a neighboring track with a distance below 10 pixels was found for at least 2 overlapping frames. In contrast, tracks were assigned to the category "noise-based fragmentation" if such a neighboring track with a distance below 10 pixels was only observed in the terminating frame. Both criteria were determined by a visual inspection of a representative amount of tracks from a representative time series and an evaluation of the reason of track termination of these tracks, combined with an evaluation of the distribution of tracking-related parameters (shown in Figure S5-7). Tracks assigned to these two “fragmentation” categories were refined as described in the main manuscript.

All ID tracks that did not fulfill any of the above conditions are added to the "unassigned" category, which therefore collects all tracks that were not automatically assigned to one of the previous categories. A visual inspection of tracks belonging to the "unassigned" category revealed that the corresponding bacteria exhibited a directed motion with a high inclination angle (with respect to the focal plane), causing them to quickly leave the focal plane. As a consequence, the intensity value in the terminating frame was significantly higher than the intensity threshold value used for the category “out of boundary” (mentioned above). Hence, as visual inspection revealed that these bacteria belong to the “out of boundary” category, all bacteria of the "unassigned" category were transferred to the “out of boundary” category.

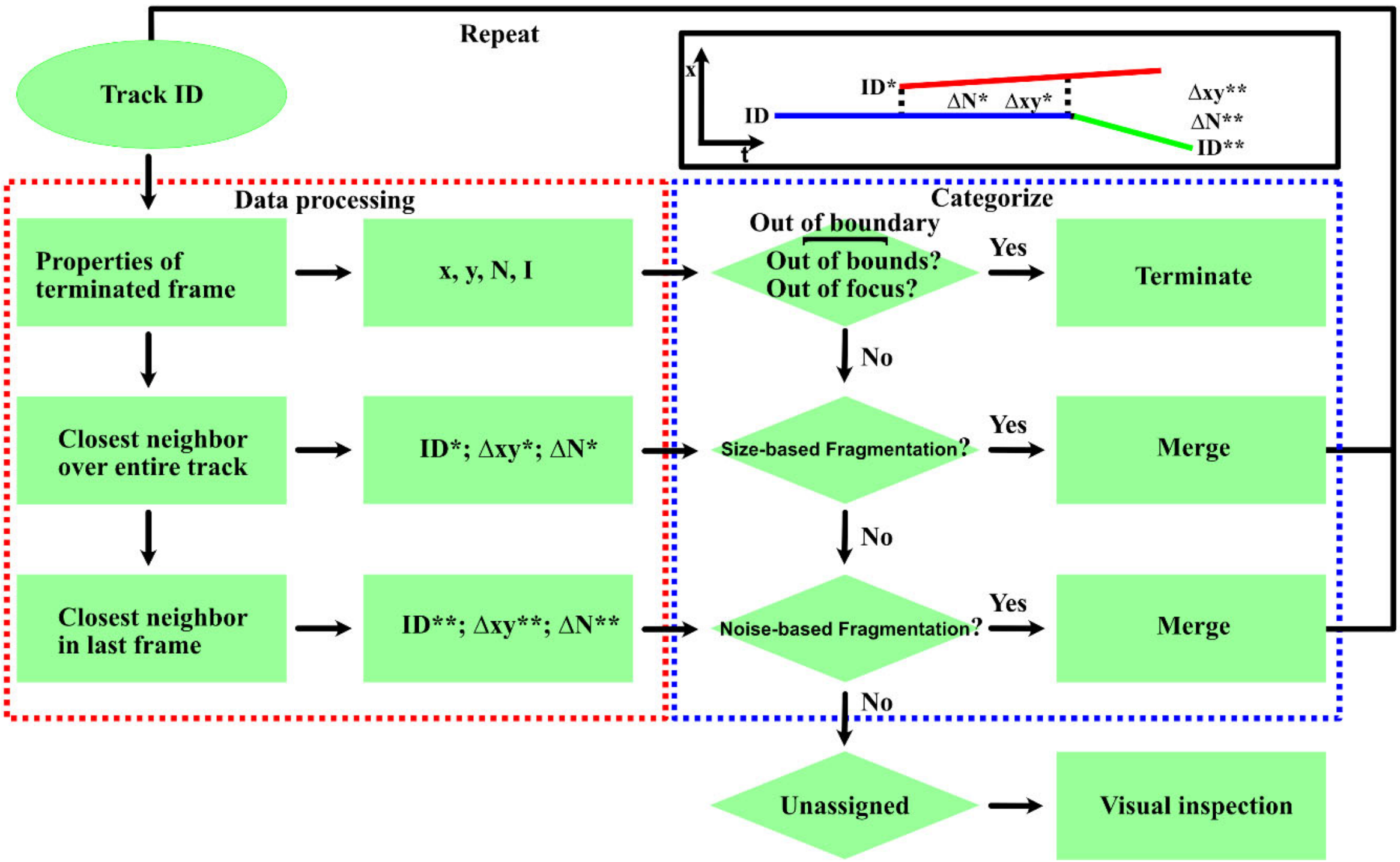

Figure S4: Analysis workflow involving the classification of tracks into different categories (according to the reason of track termination) followed by the refinement of tracks (if applicable). Each detected track is assigned an individual identifier (ID). Only tracks covering at least 5 frames are regarded in the "data processing" steps, in which various track parameters are determined: i) the lateral position (x, y) in the final frame, ii) the track length N, iii) the intensity I in the final frame. In addition, for all frames covered by the analyzed track, the distance to all remaining tracks is calculated on a frame-wise basis, allowing to identify the neighboring track that exhibits the smallest distance to the analyzed track over the entire track duration, yielding the ID of this "neighbor" (denoted by ID) as well as the observed minimum value of the lateral distance (Δxy*) and the number of overlapping frames (i.e., the temporal overlap of ID and ID*, denoted by ΔN*). Similarly, the "neighbor" with the smallest average distance Δxy** in the terminating frame can be determined (denoted as ID**) as well as the number of overlapping frames between ID and ID** (denoted as ΔN**). Using these extracted parameters, each track is assigned into one of the 3 categories: i) "out of boundary", ii) "size-based fragmentation", and iii) "noise-based fragmentation" as indicated by the flow-chart inside the blue dashed area. Tracks are assigned to the "out of boundary" category, provided that the final position is at the border of the field of view or the final intensity I drops below the threshold (see Figures S5 and S6). The remaining tracks are assigned to the "size-based fragmentation" category, provided that the minimum distance to the nearest neighbor taken over the entire track (Δxy*) falls below the average S.* Typhimurium *size (long axis ~ 10 pixel, see Figure S7) and the corresponding temporal overlap ΔN* is > 1 frame. The remaining tracks are assigned to the "noise-based fragmentation" category, provided that the minimum distance to the nearest neighbor taken in the final track (Δxy**) falls below the average S.* Typhimurium *size and the corresponding temporal overlap ΔN** equals 1 frame. Tracks, which cannot be assigned to one of these categories is assigned to the "unassigned" category, in which the reason of track termination cannot be extracted from the available parameters but requires manual inspection.*

S1.6 Measurement statistics and key motility parameters

Table S1: Overview of the total number of tracks recorded in a representative experiment for wild-type and motility-related *S*. Typhimurium mutants (as indicated). *S.* Typhimurium. The table also reports the frequency for observing track termination arising from: *i*) bacteria leaving the readout volume (out-of-boundary), *ii*) overcounting due to readout noise (noise-based fragmentation) or *iii*) overcounting due to an extended bacterial shape (size-based fragmentation).

***Salmonella* Strain:**	**Wild-type**	**Δ*fliB***	**Δ*fljC***	**Δ*fliBC***	**Wild-type glycerol 20 % w**	**Wild-type glycerol 40 % w**	**Wild-type glycerol 60 % w**	**Wild-type glycerol 80 % w**
All	25082 (100 %)	72567 (100 %	40241 (100 %)	40968 (100 %)	20245 (100 %)	15969 (100 %)	10690 (100 %)	4340 (100 %)
(i) Out-of-boundary	19760 (79 %)	54543 (75 %)	32335 (80 %)	21449 (52 %)	14718 (73 %)	10369 (65 %)	6190 (58 %)	2509 (58 %)
(ii) Noise-base Fragmentation	2126 (8 %)	5536 (8 %)	3350 (8 %)	2926 (7 %)	1967 (10 %)	1084 (7 %)	840 (8 %)	623 (14 %)
(iii) Size-based Fragmentation	3196 (13 %)	12488 (17 %)	4556 (11 %)	16593 (41 %)	3560 (17 %)	4516 (28 %)	3660 (34 %)	1208 (28 %)

Table S2: Impact of medium viscosity on the motility of wild-type *S.* Typhimurium. Shown are (if not stated otherwise) median values ± standard deviation of the indicated properties. All bacterial tracks, which covered at least 5 frames, were included in this analysis. Bacteria with $D_{\perp}$ values exceeding 1 µm²/s (0% vol.-% glycerol), 0.5 µm²/s (20 vol.-% glycerol) and 0.4 µm²/s (40 vol.-% glycerol), respectively.

**Glycerol vol.-% (wt%)**	**Viscosity[1, 2] (mPa·s, 20°C)**	$D_{\perp,active}$ **(µm²s⁻¹)**	$D_{\perp,passive}$ **(µm²s⁻¹)**	$v_{\|\|}$ **(µms⁻¹)**	**Maximum** $v_{\|\|}$ **(µms⁻¹)**	**Tracks N**	**Active [%]**
0 % pure buffer	~1.0	2.4 ± 2.0	0.38 ± 0.19	7.2 ± 7.4	39.7	8705	42
20 % (~24 %)	~2.0	0.96 ± 0.95	0.23 ± 0.09	3.1 ± 3.6	18.3	6676	25
40 % (~46 %)	~5.0	0.57 ± 3.8	0.14 ± 0.06	1.9 ± 3.4	18.4	1811	6
60 % (~65%)	~15.5	n.d.	0.08 ± 1.20	n.d.	n.d.	1005	0
80 % (~83 %)	~88.0	n.d.	0.03 ± 0.15	n.d.	n.d.	290	0

S2. Distributions of properties related to bacterial tracking

The following plots correlate various parameters determined by single particle tracking of *S.* Typhimurium (as indicated in the corresponding plots).

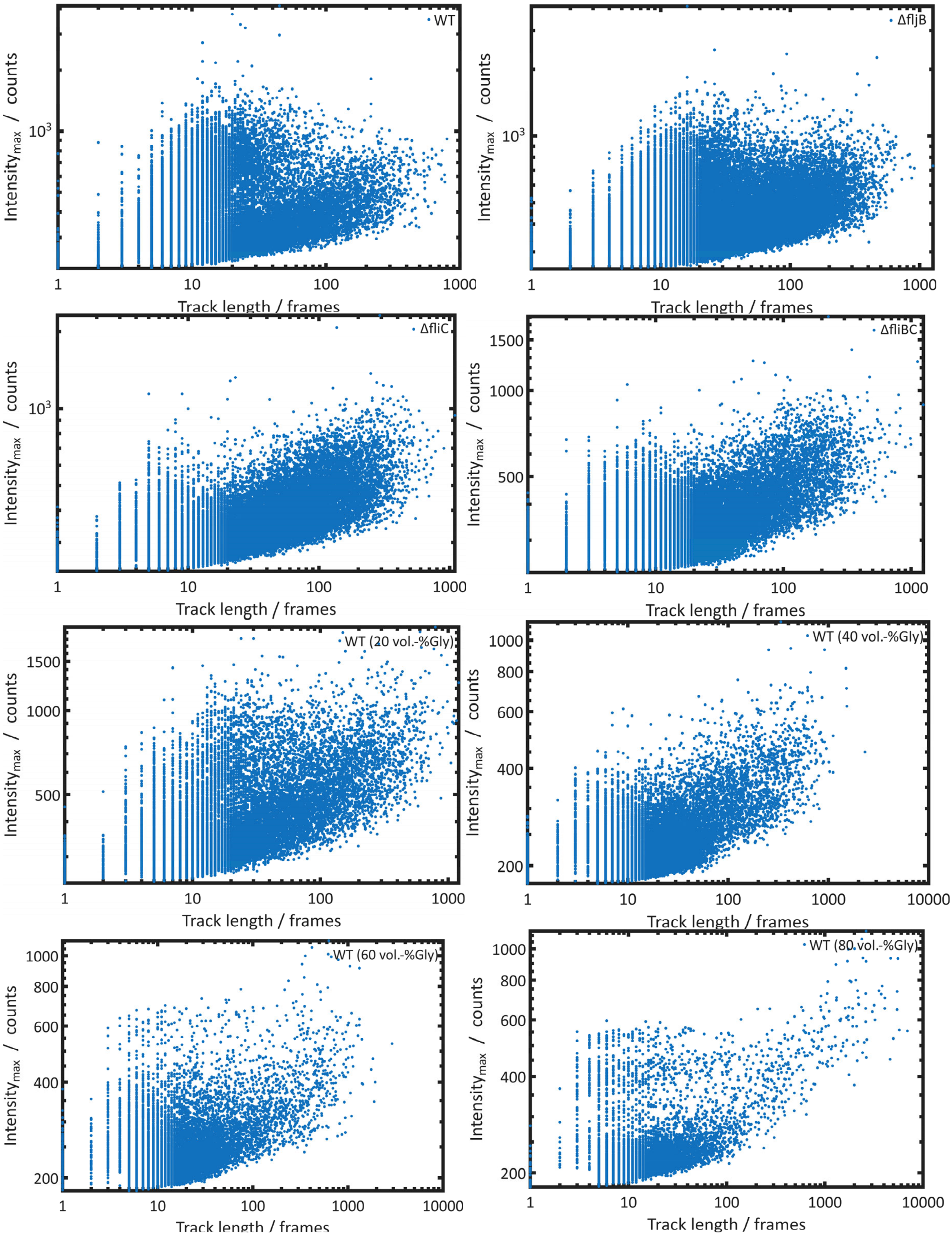

Figure S5: Scatter plots correlating track length with the maximum intensity observed for the corresponding bacterium (each dot corresponds to an individual bacterium). Both axes are plotted on a logarithmic scale.

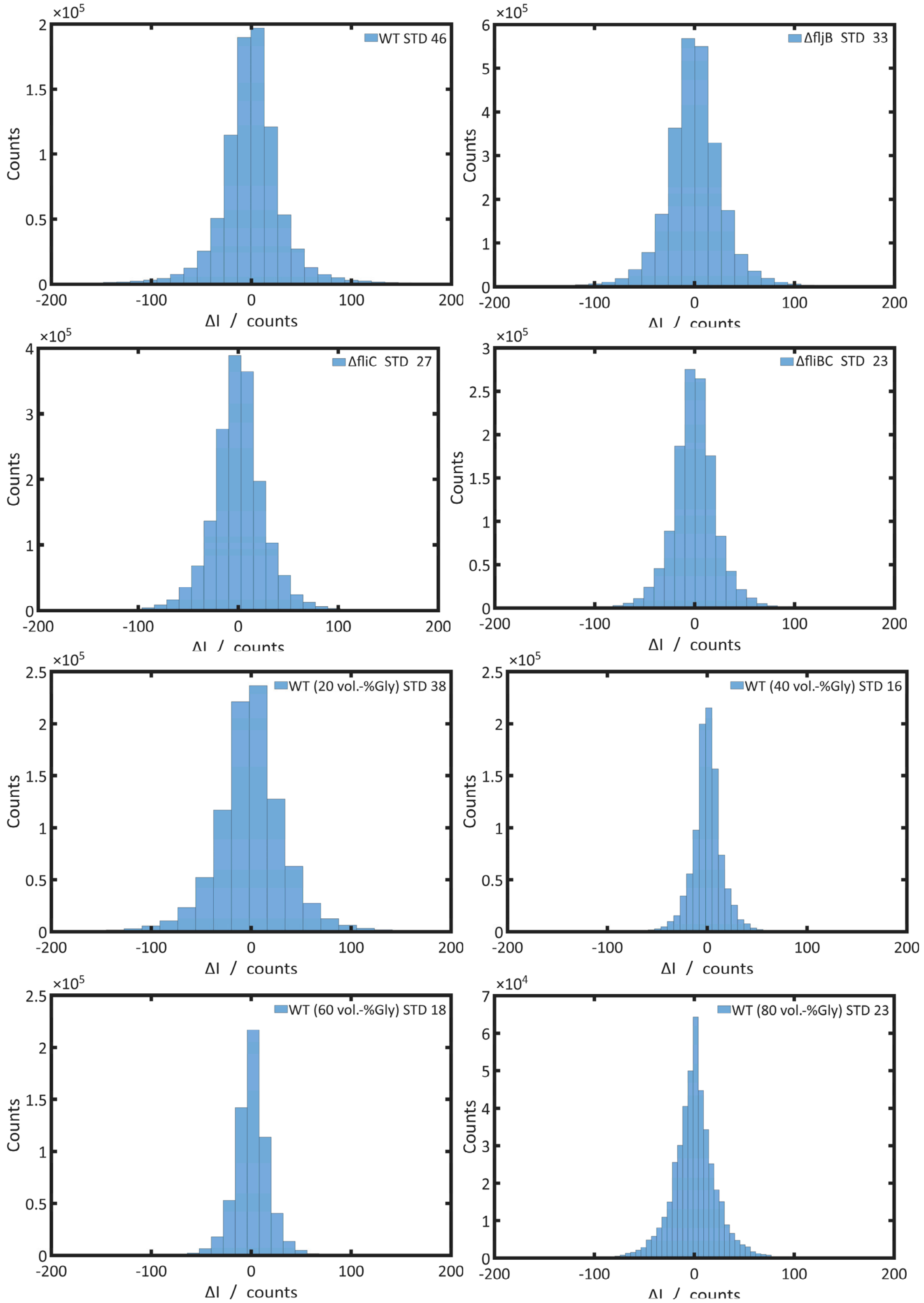

Figure S6: Distribution of the change of the intensity value of individual bacteria observed between consecutive frames. These distributions exhibit (on average) a standard deviation on the order of 35 counts.

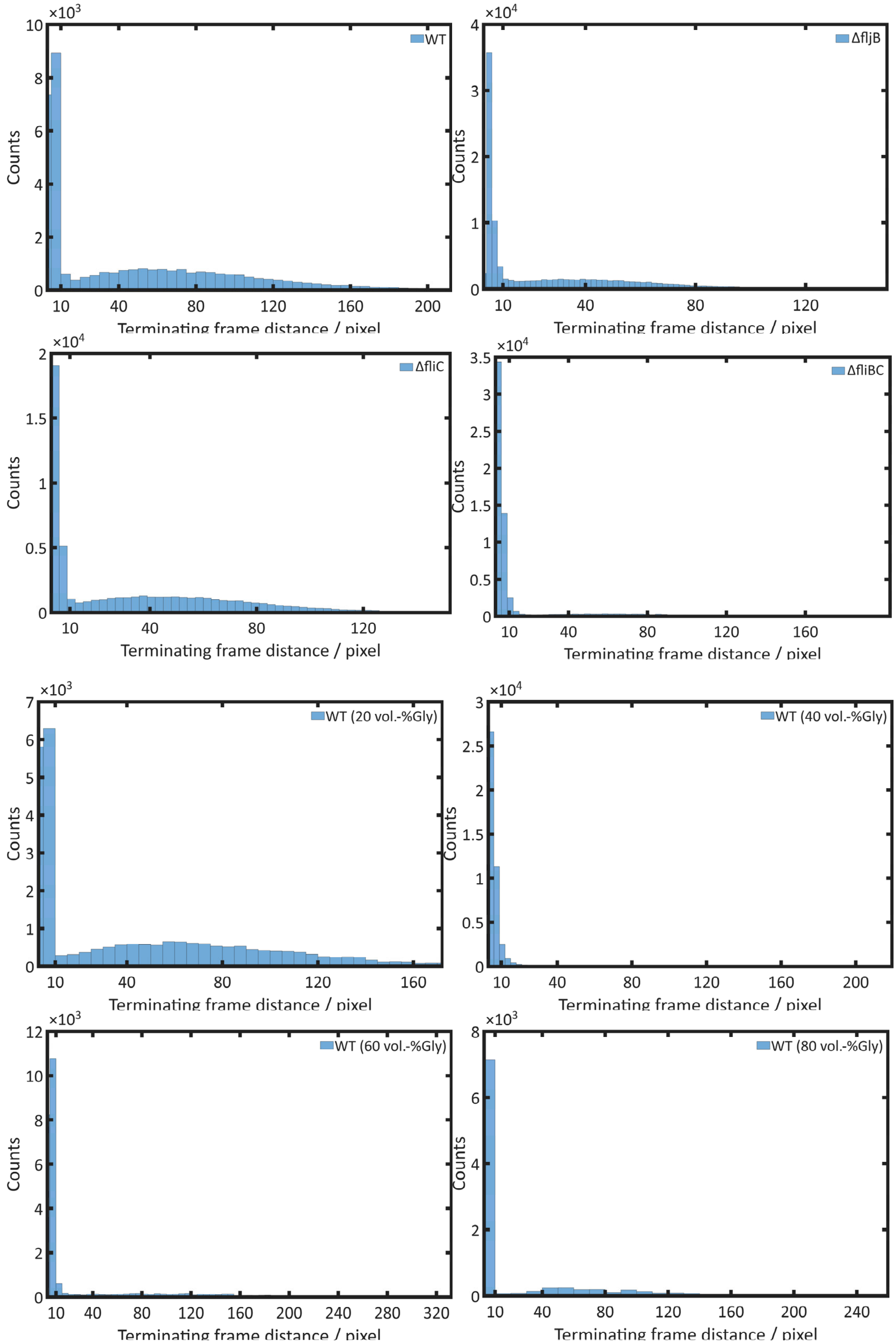

Figure S7: For each track, the distance to the nearest neighbor was determined in the final frame of the track. The resulting histograms (shown above) exhibit one sharp and one broad peak, which are separated by ~10 pixels (independent of strain or experimental condition). Visual inspection revealed that the sharp peak is generated by noise- and size-based track fragmentation (consistent with the notion that 10 pixels correspond to ~2 µm, i.e., half of the S. Typhimurium *length), while the broad peak corresponds to other reasons of track termination (e.g., leaving the field of view).*

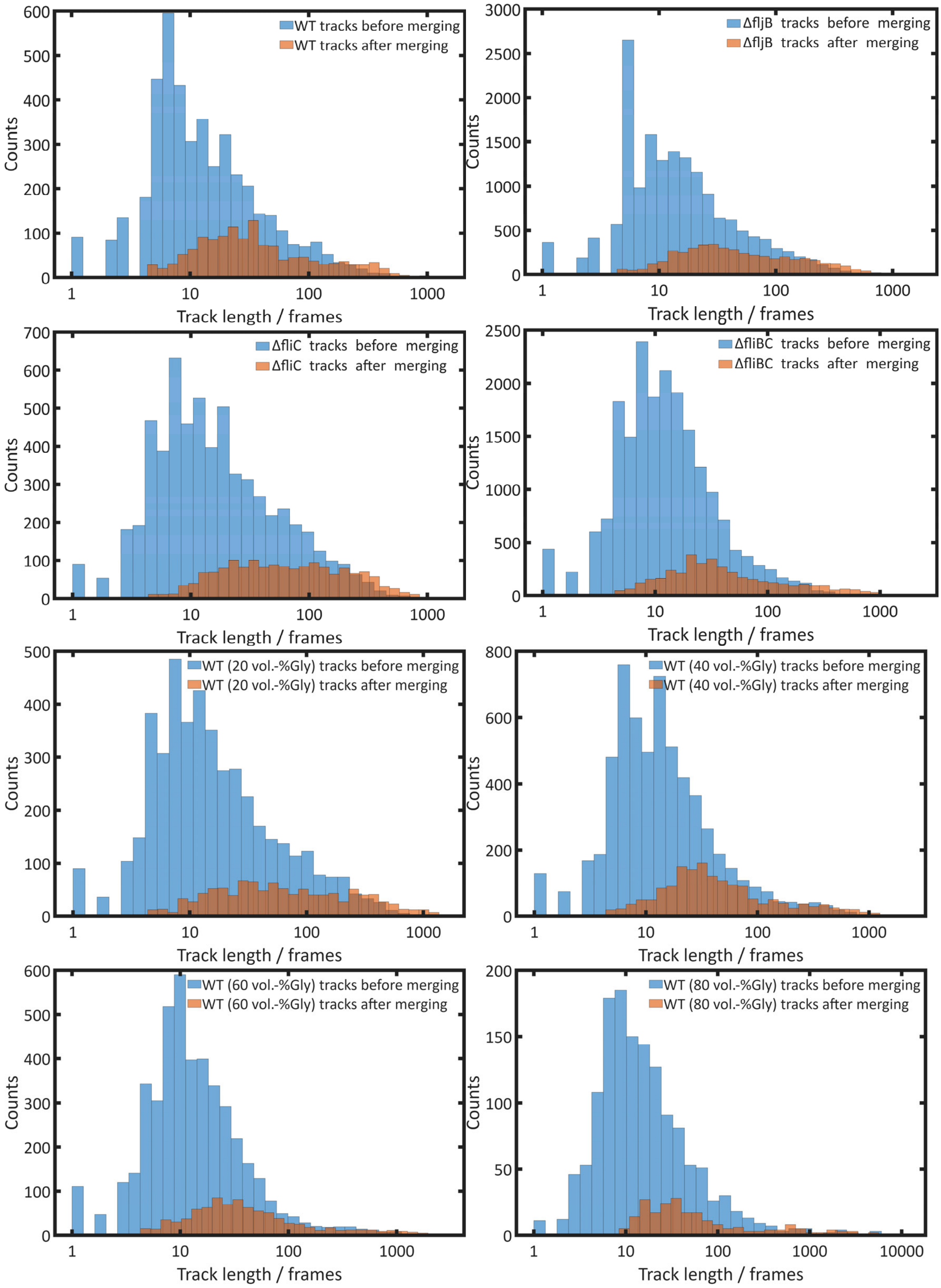

Figure S8: Track length of tracks belonging to the category "size-based fragmentation". The corresponding tracks are merged and the track length distributions (ending minus starting frame number) were determined prior to and after this refinement. With 23 ms between consecutively frames, so consequently 100 frames represent a trajectory over 2.3 s.

S3. Validation of size extraction procedure

To validate the extraction of the short and long axis of rod-like structures using a centroid approach, synthetic (*i.e.*, simulated) time series showing rods, that exhibit Brownian diffusion, were generated and analyzed using our home-made SPT analysis workflow. In these simulations, rods with a long and short axis of 2.5 and 1 µm, respectively, were generated, corresponding to an aspect ratio of 2.5. The SPT analysis yielded median values of long and short axis of 2.4 and 1 µm, respectively, which differed less than 6% from the values used in the simulation.

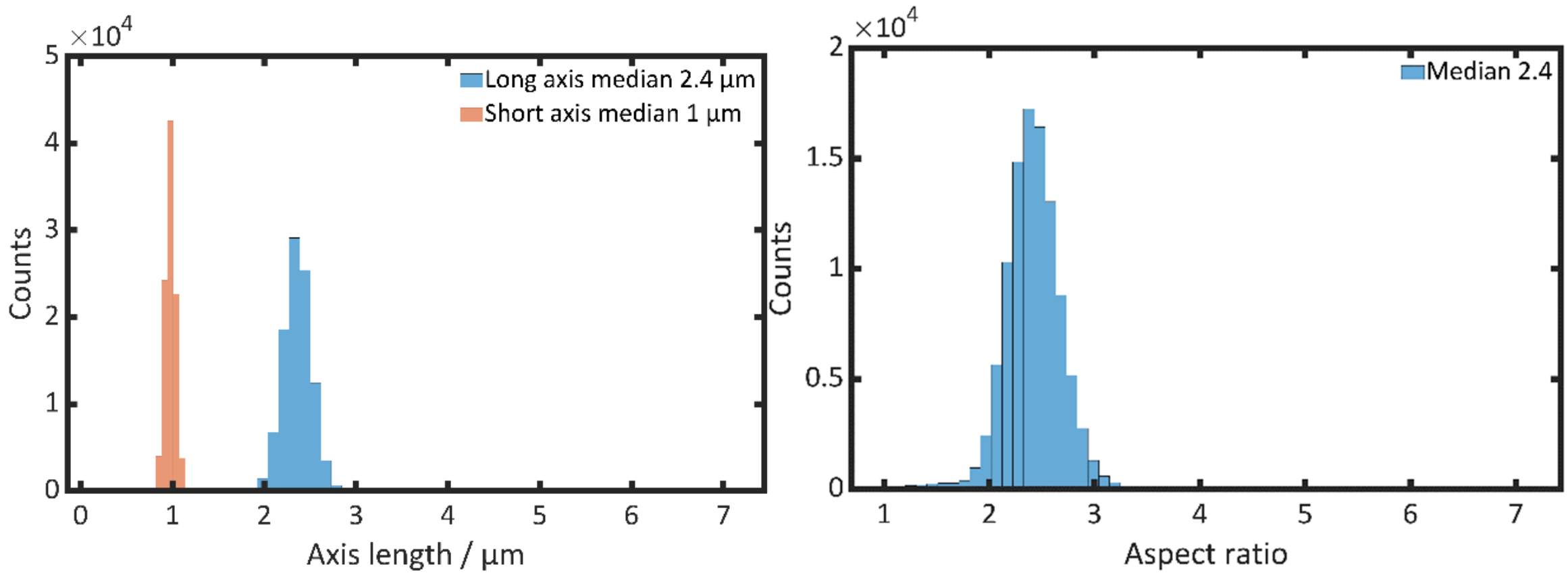

Figure S9: Long and short axis (left) and aspect ratio (right) of simulated rods exhibiting 2D Brownian diffusion. The distributions are centered around ~ 1 µm (short axis), ~ 2.4 µm (long axis), and ~ 2.4 (aspect ratio) and agree within 6% with the input values (1 µm, 2.5 µm, and 2.5, respectively).

S4. Diffusion coefficient distributions

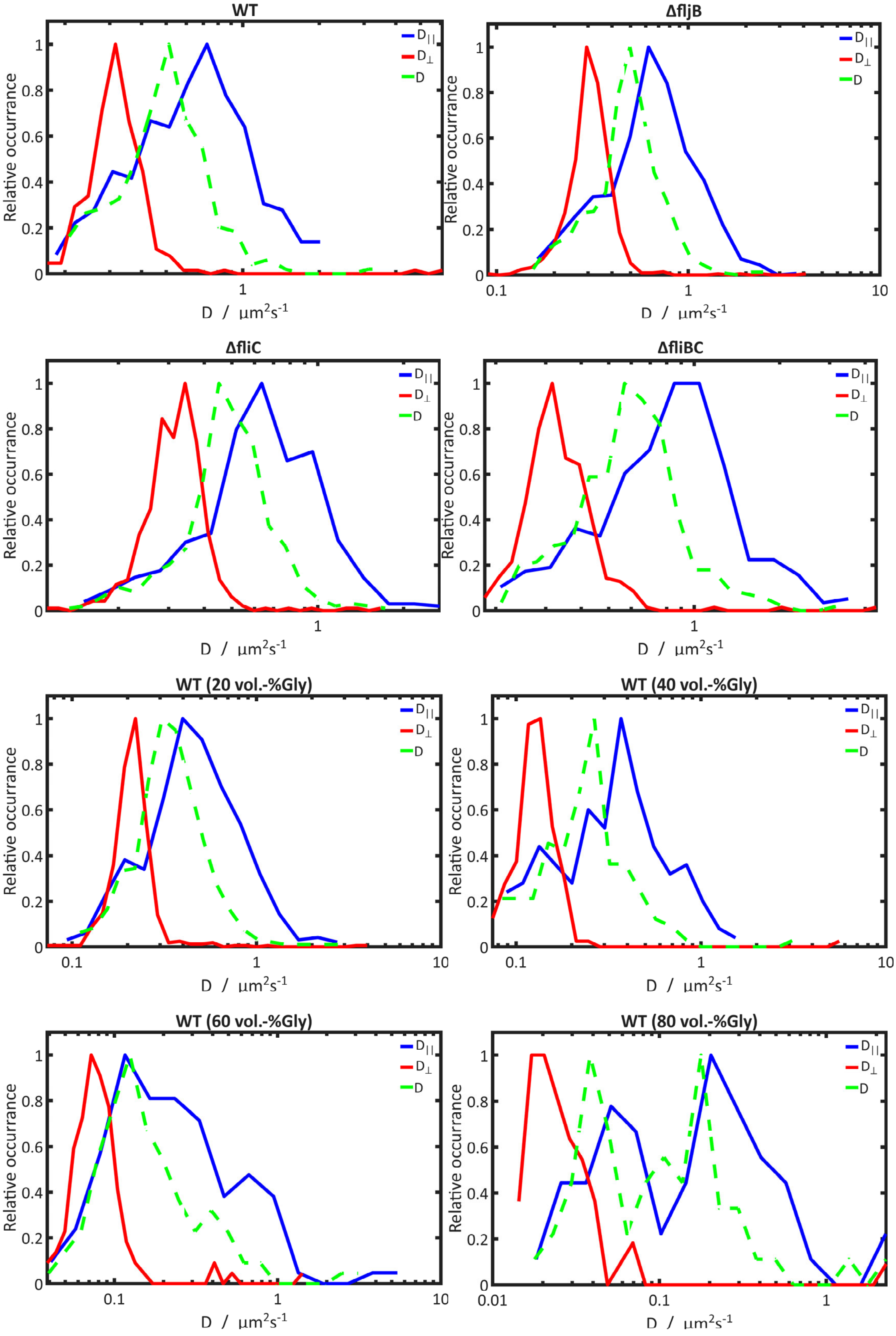

Figure S10: Distributions of the rates of short-term positional fluctuations of the bacterial center position extracted for random motion parallel ($D_{\parallel}$, blue) and perpendicular ($D_{\perp}$,red) to the bacterial figure axis or without accounting for bacterial anisotropy (D,green). (Track inclusion criteria: inactive bacterial population, track length > 50, average aspect ratio ≥ 1.75.)

S5N. Mean squared displacement analysis

In this study, the short-term fluctuations of the bacterial center position are quantified parallel and perpendicular to the bacterial long axis using the equations

$$D_{\parallel} = \frac{var(\Delta r_{\parallel})}{2\Delta t} \quad \text{and} \quad D_{\perp} = \frac{var(\Delta r_{\perp})}{2\Delta t} \qquad \text{(S1)}$$

in which $var(x) =< x^2 > - < x >^2$ denotes the variance calculated over an entire track, $\Delta r_{\parallel}$ and $\Delta r_{\perp}$ the (one-dimensional) displacements parallel and perpendicular to the long axis and Δt the time period between two consecutive frames. For passively moving (*i.e.*, diffusing) bacteria, which do not exhibit systematic drifts in $\Delta r_{\parallel}$ or $\Delta r_{\perp}$, Eq. S1 is equivalent to

$$D_{\parallel} = \frac{\langle \Delta r_{\parallel}^2 \rangle}{2\Delta t} \quad \text{and} \quad D_{\perp} = \frac{\langle \Delta r_{\perp}^2 \rangle}{2\Delta t} \qquad \text{(S2)}$$

which is more commonly employed in SPT analysis than Eq. S1.

We confirmed the equivalence for passively moving (*i.e.*, diffusing) bacteria by calculating single-cell mean squared displacement (MSD) curves for fluctuations of the bacterial center position parallel and perpendicular to the bacterial long axis, $MSD_{\parallel}$ and $MSD_{\perp}$, according to

$$MSD_{\parallel}(j \cdot \Delta t) = \frac{1}{N-j} \sum_{n=1}^{N-j} \left(\Delta r_{n,\parallel}(j \cdot \Delta t) \right)^2 \quad \text{and} \qquad \text{(S3)}$$

$$MSD_{\perp}(j \cdot \Delta t) = \frac{1}{N-j} \sum_{n=1}^{N-j} \left(\Delta r_{n,\perp}(j \cdot \Delta t) \right)^2$$

with $j \cdot \Delta t$ denoting the lag time (in multiples of the frame-to-frame period Δt), at which the MSD is evaluated, $\Delta r_{n,\parallel}(j \cdot \Delta t)$ and $\Delta r_{n,\perp}(j \cdot \Delta t)$ the displacements parallel and perpendicular to the long axis calculated for all frames of the track, which are separated by the lag time $j \cdot \Delta t$, and $N - j$ the number of such frame pairs of the analysed track. In this analysis, only tracks were included that contained at least 50 frames with information on the bacterial orientation. As actively moving bacteria left the observation volume much faster, the analysed population almost exclusively consisted of passively diffusing bacteria.

The obtained MSD curves (Figure S11) showed a linear dependence of the lag time on the single-cell as well ensemble-averaged level. We determined the slope of the ensemble-averaged MSD curves using[3]

$$MSD_i(j \cdot \Delta t) = 2D_i^{\mathrm{MSD}}(j - 2r)\Delta t + 2\sigma_i^2, \quad i \in \{\parallel, \perp\} \qquad \text{(S4)}$$

in which σ is connected to localization noise and r is the ratio of camera exposure time Δt_{exp} to Δt and accounts for motional blur: $r = \Delta t_{exp}/\Delta t$. Fitting Eq. S4 yields $D_{\perp}^{\mathrm{MSD}} = 0.38$ µm²/s and $D_{\parallel}^{\mathrm{MSD}} = 0.56$ µm²/s. These values are in good agreement with the median values of the D-distributions shown in Figure 4C (main manuscript), which are located at 0.32 and 0.64 µm²/s, respectively. Setting $r = 0$ during fitting does not affect the D^{MSD} values, indicating that the impact of motional blur can be neglected. In addition, the linearity of the MSD curves and the good agreement of the

D-values with theoretical estimates for the diffusion of rod-like objects indicates that for passively diffusing bacteria, $D_{\parallel}$ and $D_{\perp}$ correspond to the long-term diffusion coefficients. For actively moving bacteria, this is not the case as their long-time diffusion coefficients depends on the characteristic times of run and tumble periods as well as the velocity during run period and thus not on short-term random fluctuations of the bacterial center position quantified by Eq. S1.

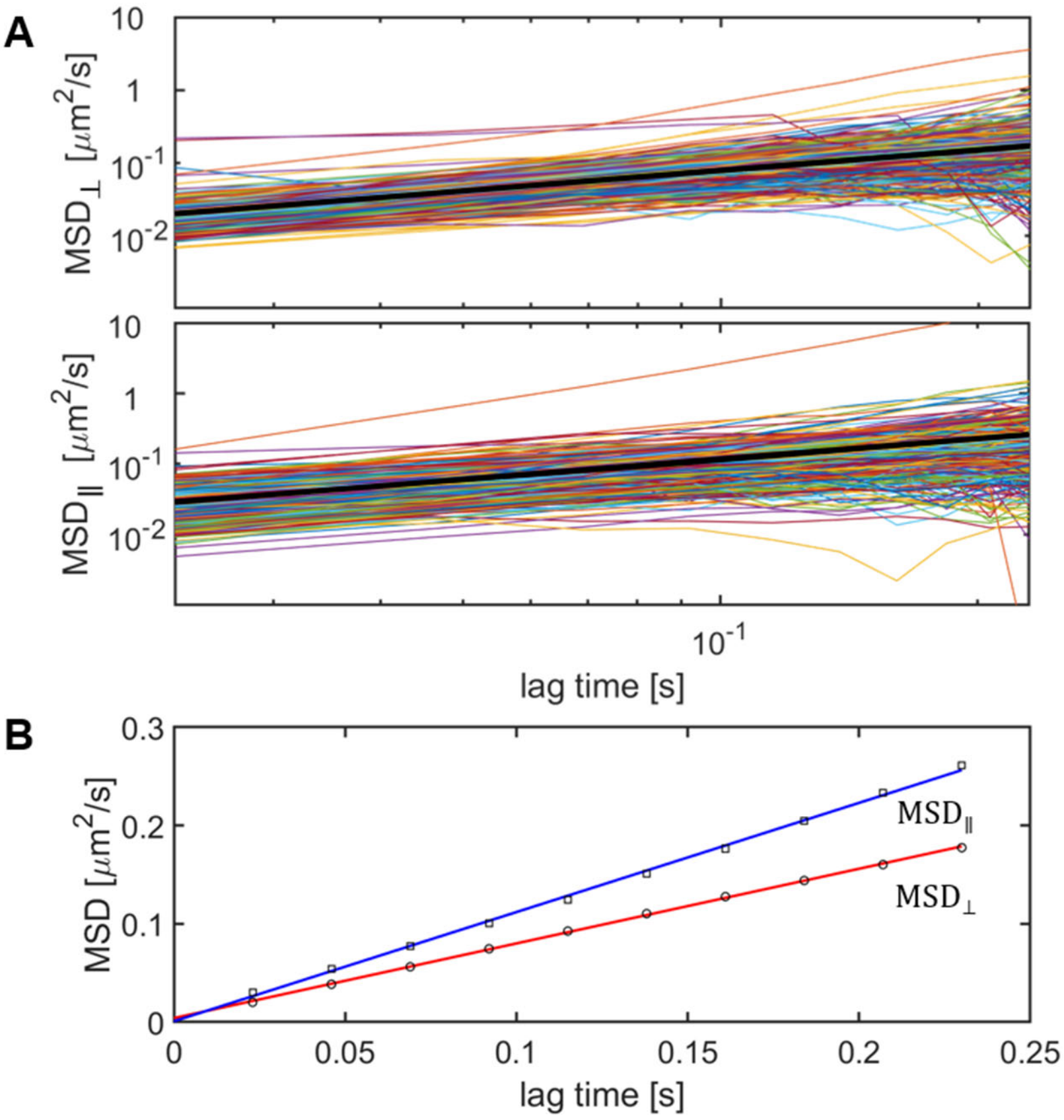

Figure S11: $MSD_{\parallel}$ *and* $MSD_{\perp}$ *curves for passively moving Salmonella Typhimurium WT. Panels a and b show the MSD curves on the single-cell level (colored curves), while c shows ensemble-averaged* $MSD_{\parallel}$ *and* $MSD_{\perp}$ *curves (i.e., the mean average of all curves shown in a and b, respectively). For comparison, the ensemble-averaged MSD curves have been added as black curves in a and b, whereas the black lines in c are fits of Eq. A1 to the corresponding MSD curve. In these calculations, all bacterial tracks have been included that contained at least 50 observations of bacterial orientation. As these tracks cover at least 50 frames (i.e., a minimum observation time > 1.2 s), they correspond almost exclusively to passively moving bacteria.*

S5. Opening angle

S5.1. Opening angle distributions

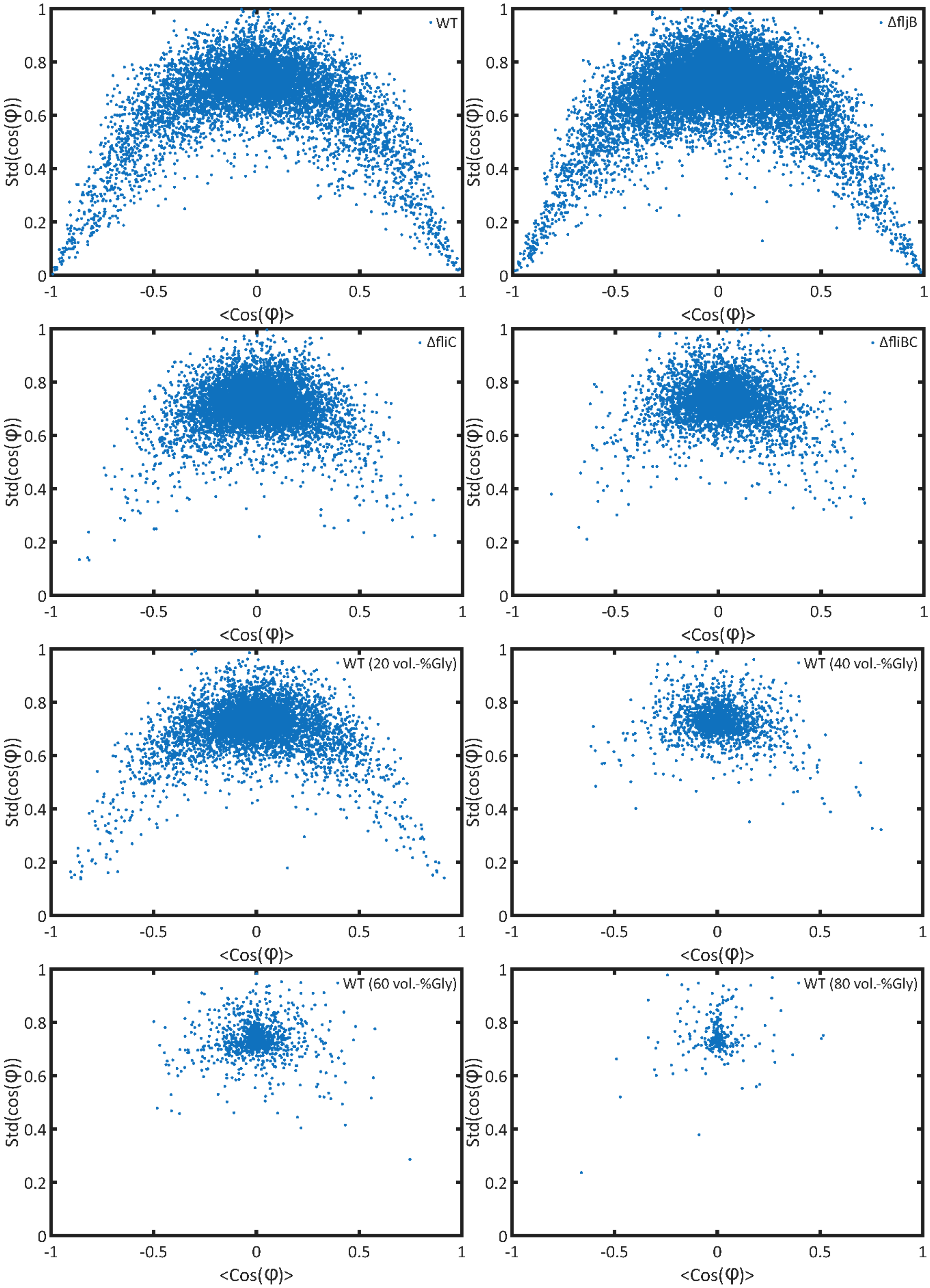

Figure S12: Scatter plots correlating the average value and standard deviation of the opening angle φ (enclosed between bacterial displacement and figure axis). (Inclusion criterium: track length > 5.)

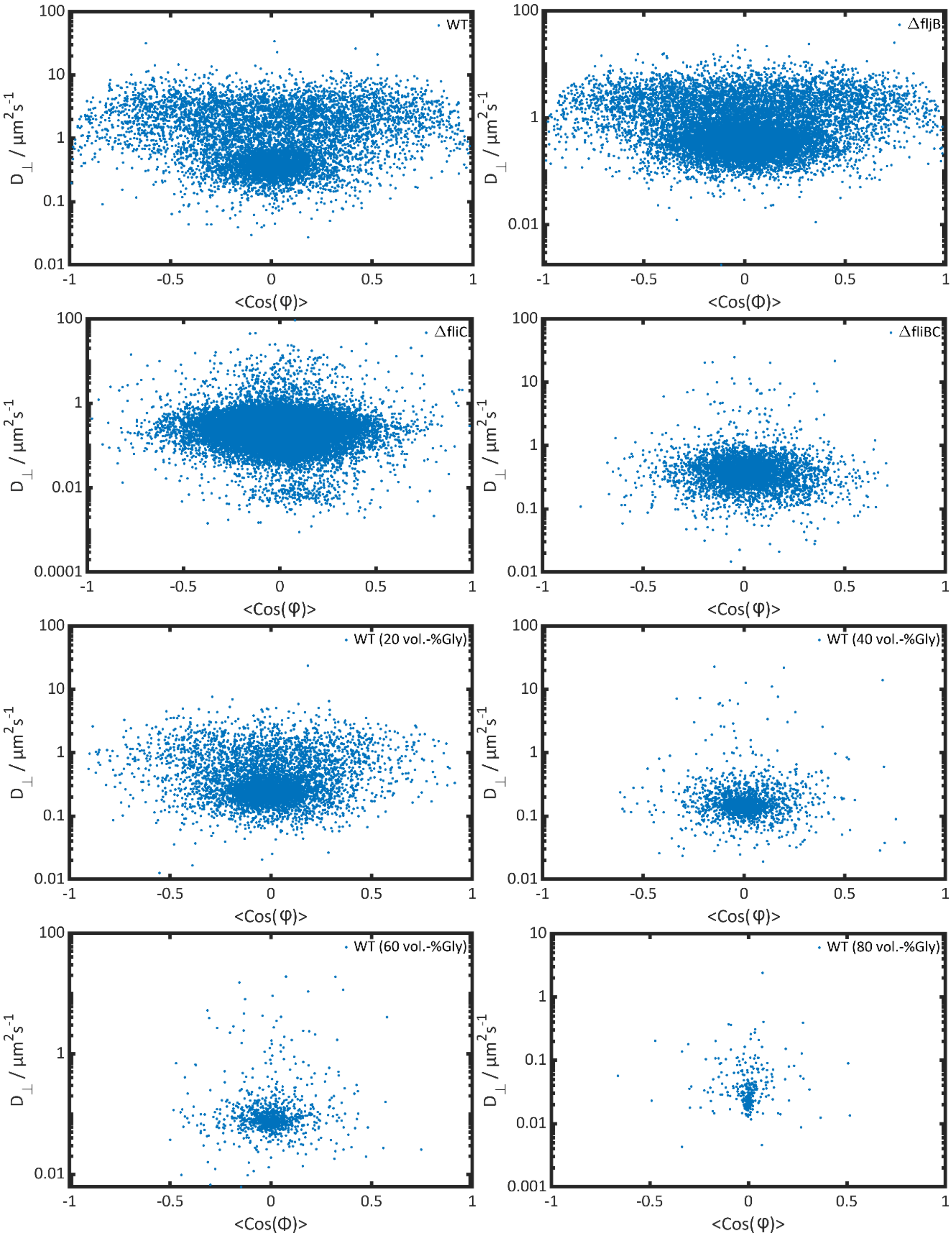

Figure S13: Scatter plots correlating $D_{\perp}$ and standard deviation of the opening angle φ (enclosed between bacterial displacement and figure axis). (Inclusion criterium: track length > 5.)

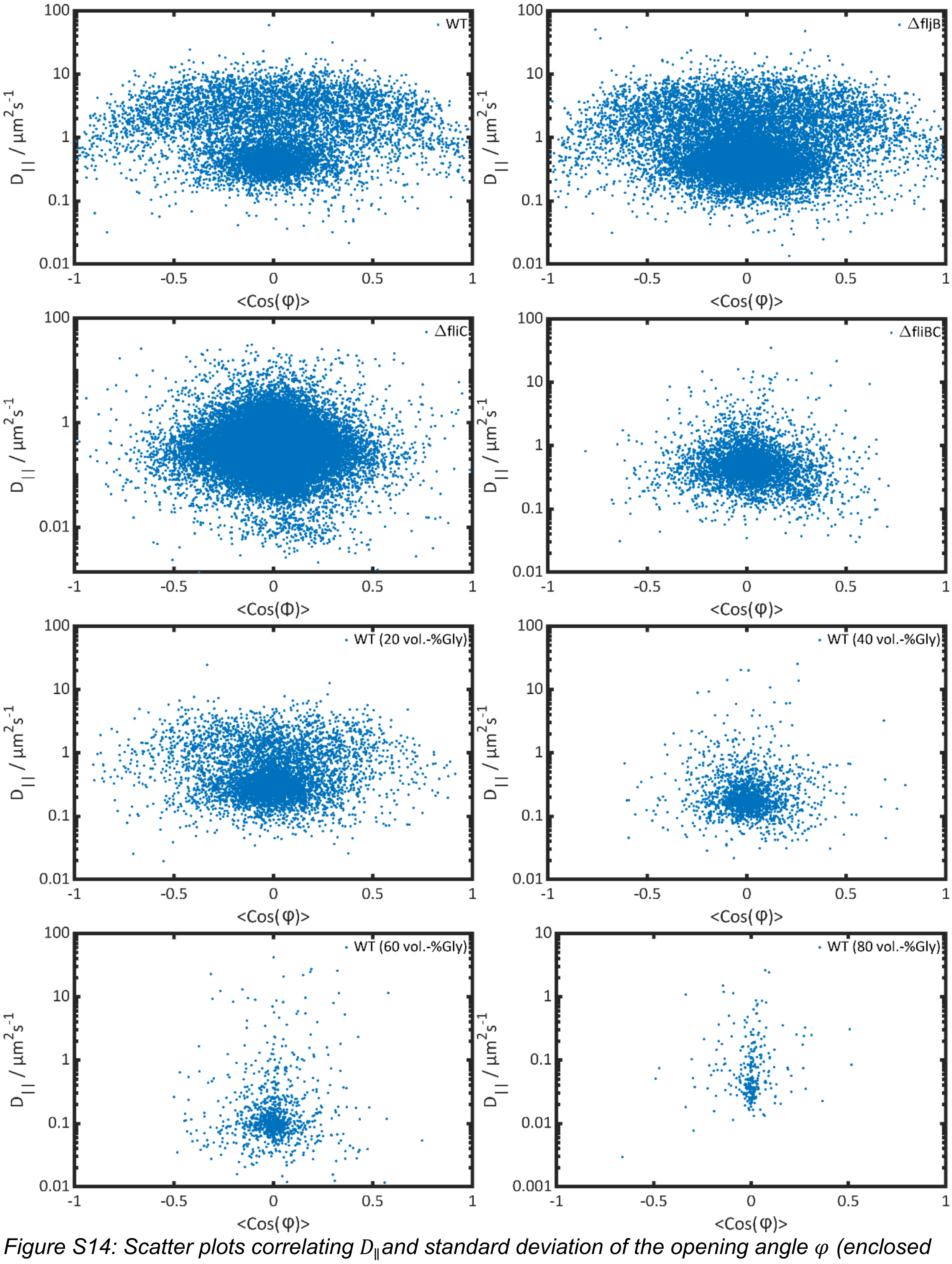

Figure S14: Scatter plots correlating $D_{\parallel}$ and standard deviation of the opening angle φ (enclosed between bacterial displacement and figure axis). (Inclusion criterium: track length > 5.)

S5.2. Precession-type motion of actively moving *S*. Typhimurium

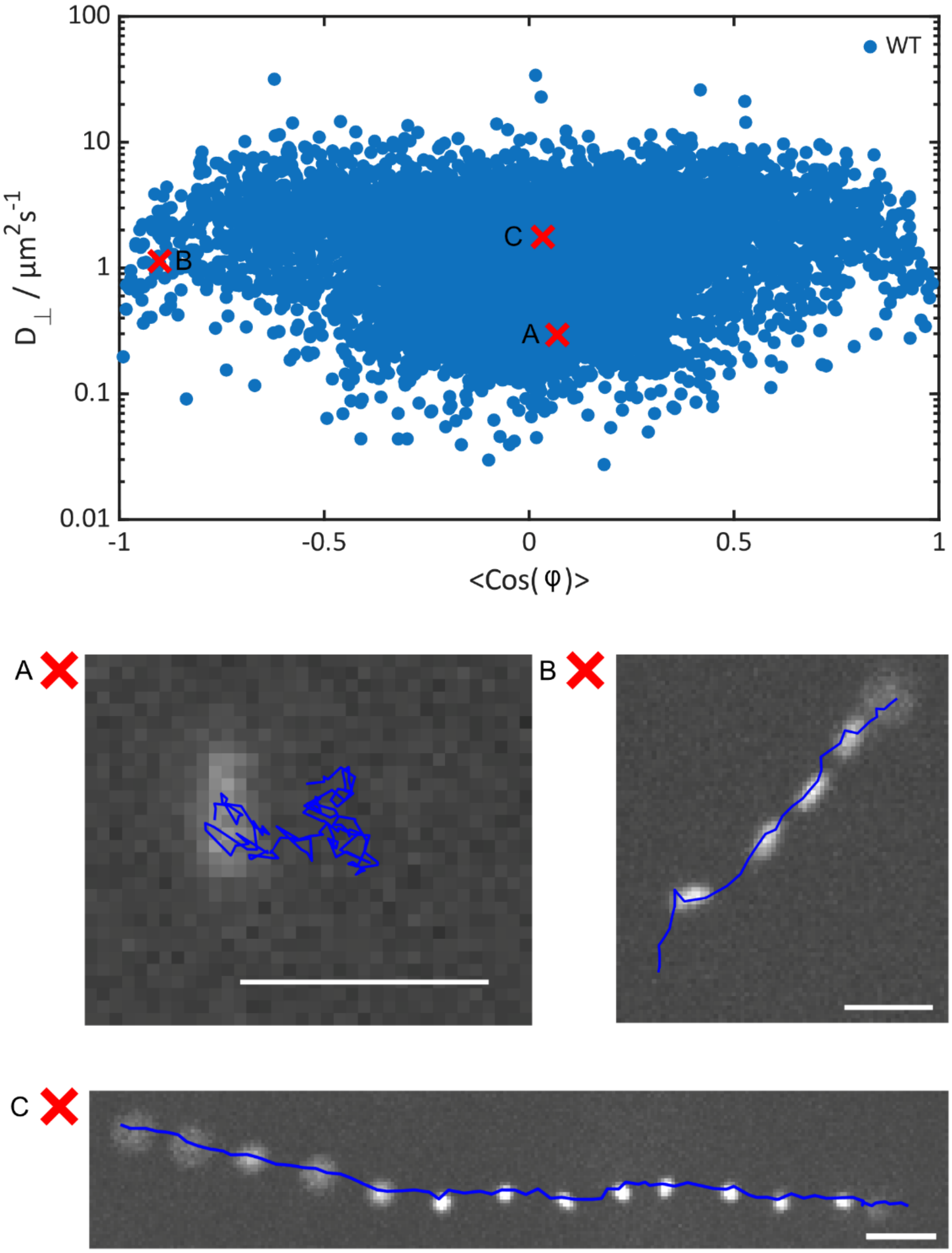

Figure S15: Representative examples of passively (A) and actively (B, C) moving bacteria (imaged using fluorescence microscopy at 43 fps) that exhibit low (A, C) and high (B) alignment between the bacterial long axis and displacement. Track C is covered in Supporting Video S1. Scale bar: 4 µm.

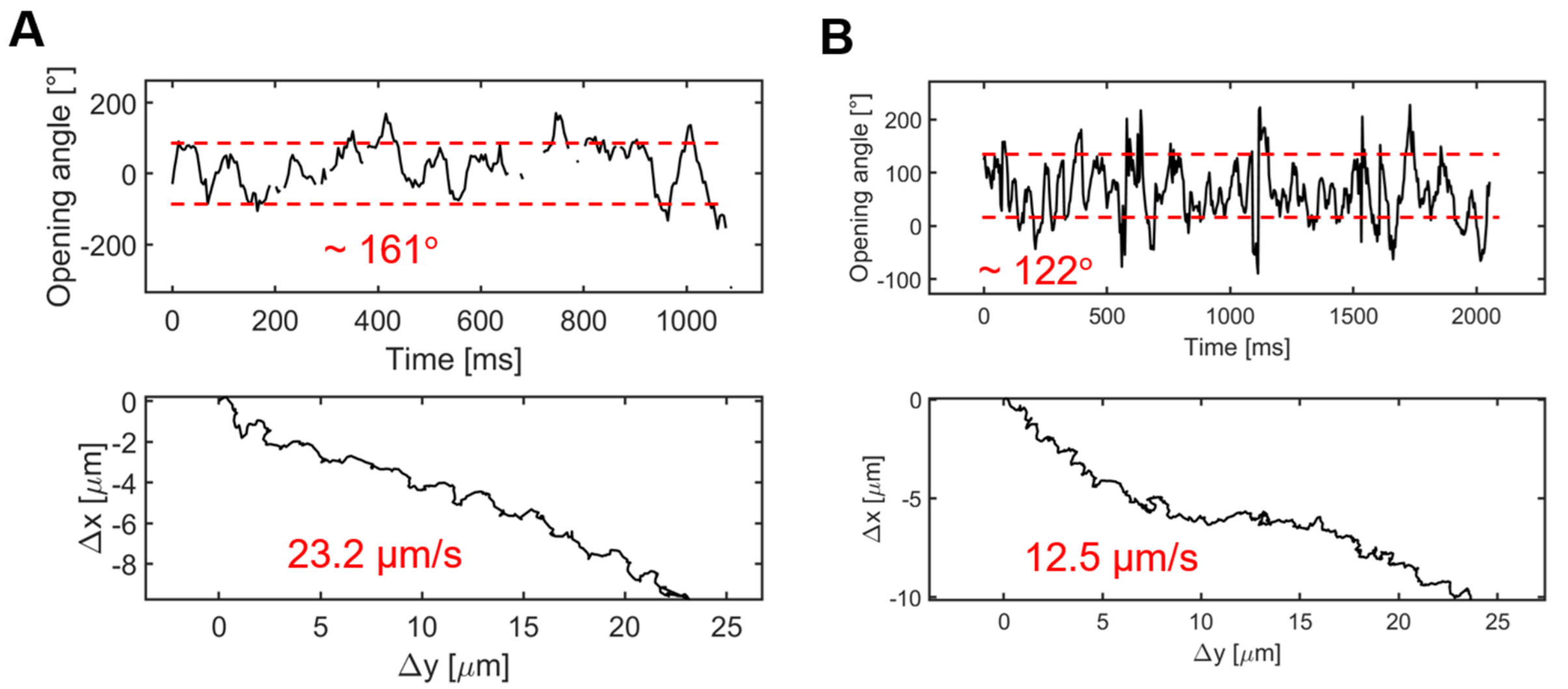

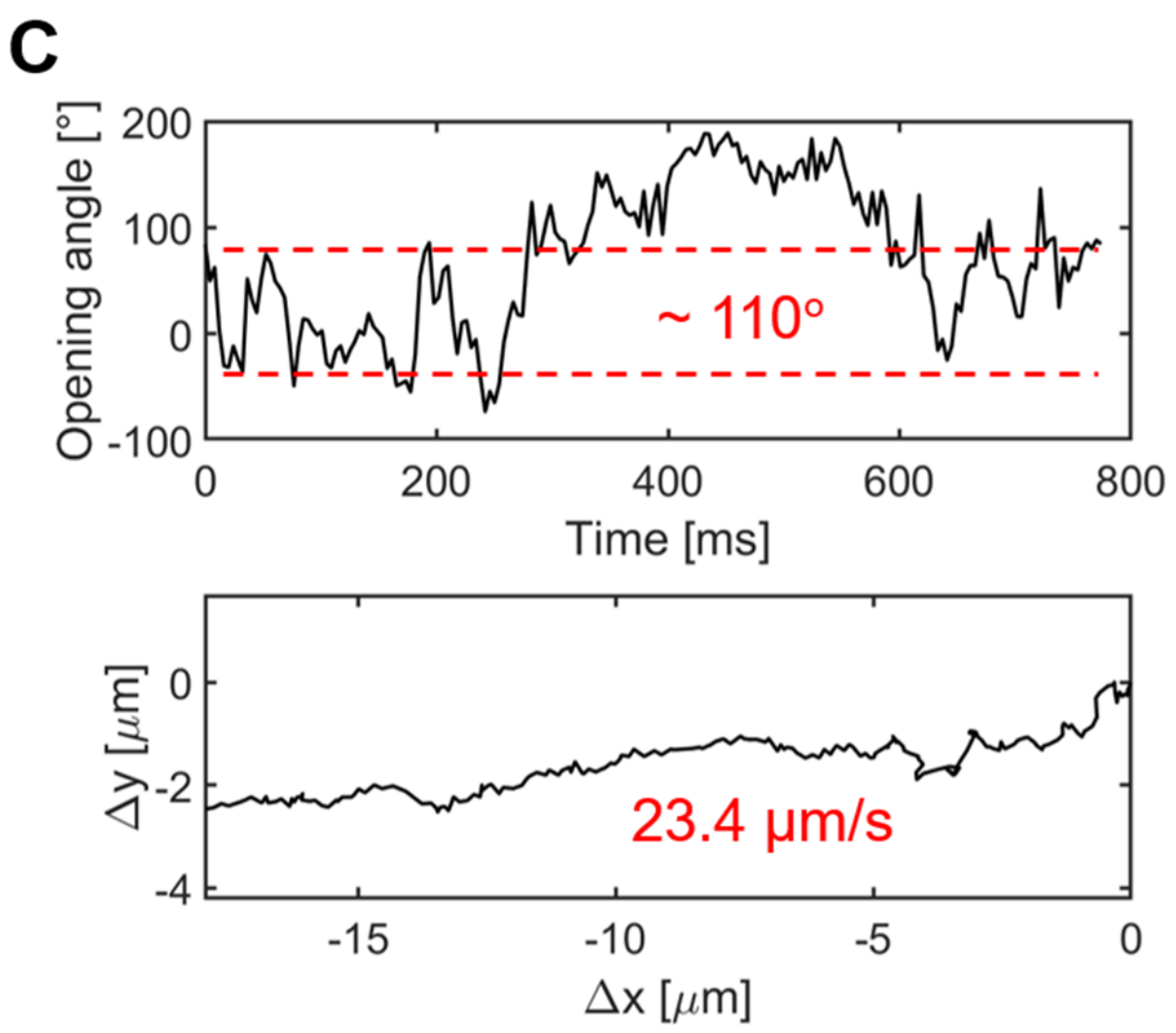

Figure S16: Representative examples for tracks showing the center position of individual, actively moving S. Typhimurium WT (imaged using fluorescence microscopy at 250 fps). Shown are the time-dependent opening angle (top panels) and the bacterial track (bottom panels). Imaged with higher temporal resolution reveals that S. Typhimurium WT can exhibit a precession-type motional pattern as indicated by the helical shape of the tracks and a notable the oscillation of the bacterial orientation. Most of the observation time, the opening angle oscillates within a corridor (dashed lines). The width of this corridor therefore equals to twice the amplitude of the oscillation. Videos showing the raw image series have been added as Supporting Videos S4-S6.

S5.3. Velocity distributions

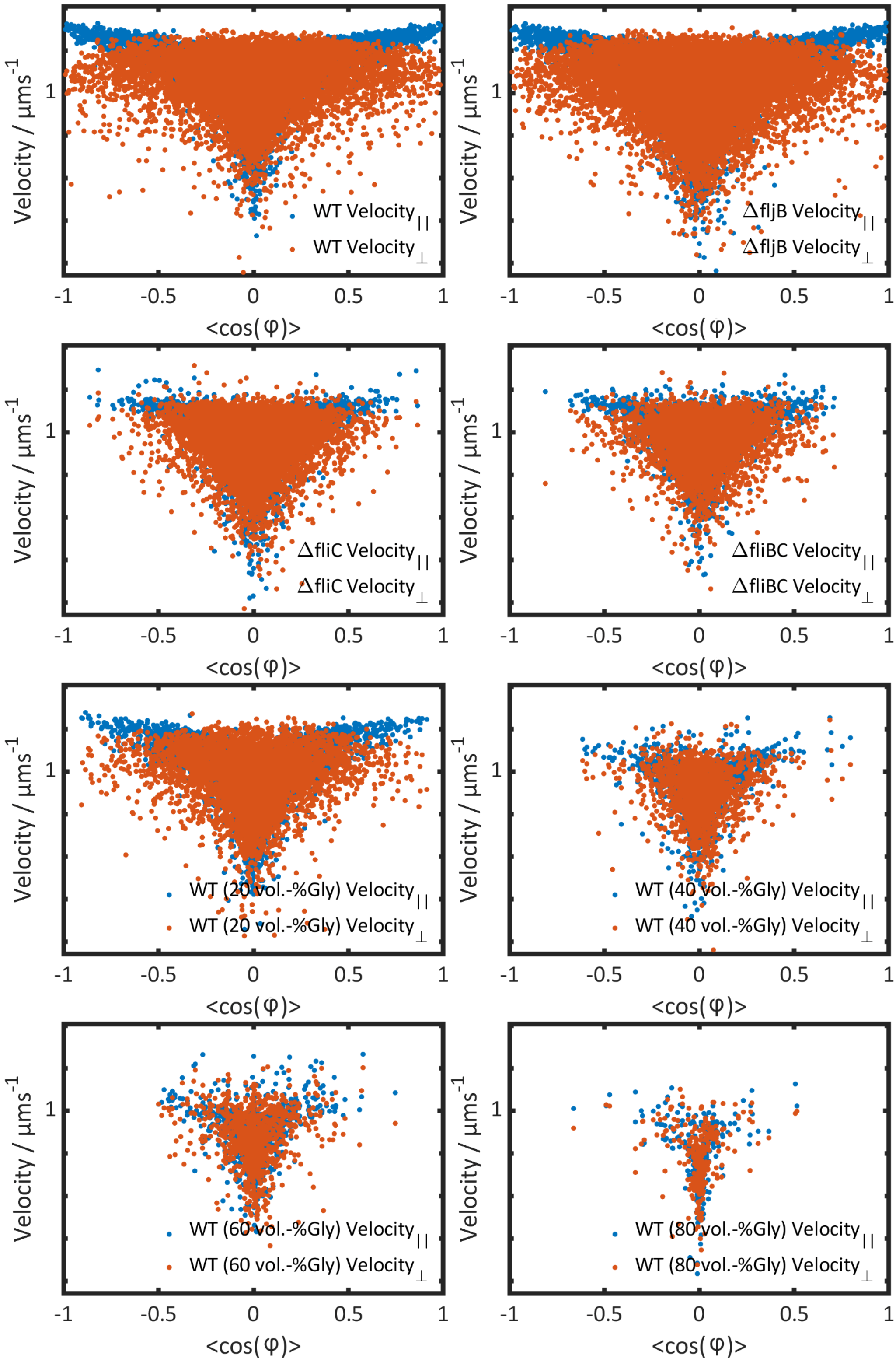

Figure S17: Scatter plots correlating the frame-to-frame velocity perpendicular ($v_{\perp}$, orange) and parallel ($v_{\parallel}$, blue) to the bacterial figure axis versus the standard deviation of the opening angle φ (enclosed between the bacterial displacement and figure axis). (Inclusion criterium: track length $>$ 5.)

S6. References

(1) Mastromarino, S.; Rook, R.; De Haas, D.; Verschuur, E. D. J.; Rohde, M.; Kloosterman, J. L. An ultrasonic shear wave viscometer for low viscosity Newtonian liquids. *Meas Sci Technol* **2021**, *32* (12). DOI: 10.1088/1361-6501/ac200f.
(2) Sheely, M. L. Glycerol Viscosity Tables. *Industrial & Engineering Chemistry* **1932**, *24* (9), 1060-1064. DOI: 10.1021/ie50273a022.
(3) Berglund, A. J. Statistics of camera-based single-particle tracking. *Phys Rev E Stat Nonlin Soft Matter Phys* **2010**, *82* (1 Pt 1), 011917. DOI: 10.1103/PhysRevE.82.011917 From NLM Medline.
